# School closure policies at municipality level for mitigating influenza spread: a model-based evaluation

**DOI:** 10.1186/s12879-016-1918-z

**Published:** 2016-10-18

**Authors:** Constanze Ciavarella, Laura Fumanelli, Stefano Merler, Ciro Cattuto, Marco Ajelli

**Affiliations:** 1 Bruno Kessler Foundation, Via Sommarive 18, 38123 Trento, Italy; 2 ISI Foundation, Turin, Italy

**Keywords:** Influenza, Mitigation, Targeted school closure, Computational modeling

## Abstract

**Background:**

Nearly every year Influenza affects most countries worldwide and the risk of a new pandemic is always present. Therefore, influenza is a major concern for public health. School-age individuals are often the most affected group, suggesting that the inclusion in preparedness plans of school closure policies may represent an option for influenza mitigation. However, their applicability remains uncertain and their implementation should carefully be weighed on the basis of cost-benefit considerations.

**Methods:**

We developed an individual-based model for influenza transmission integrating data on sociodemography and time use of the Italian population, face-to-face contacts in schools, and influenza natural history. The model was calibrated on the basis of epidemiological data from the 2009 influenza pandemic and was used to evaluate the effectiveness of three reactive school closure strategies, all based on school absenteeism.

**Results:**

In the case of a new influenza pandemic sharing similar features with the 2009 H1N1 pandemic, gradual school closure strategies (i.e., strategies closing classes first, then grades or the entire school) could lead to attack rate reduction up to 20–25 % and to peak weekly incidence reduction up to 50–55 %, at the cost of about three school weeks lost per student. Gradual strategies are quite stable to variations in the start of policy application and to the threshold on student absenteeism triggering class (and school) closures. In the case of a new influenza pandemic showing different characteristics with respect to the 2009 H1N1 pandemic, we found that the most critical features determining the effectiveness of school closure policies are the reproduction number and the age-specific susceptibility to infection, suggesting that these two epidemiological quantities should be estimated early on in the spread of a new pandemic for properly informing response planners.

**Conclusions:**

Our results highlight a potential beneficial effect of reactive gradual school closure policies in mitigating influenza spread, conditioned on the effort that decision makers are willing to afford. Moreover, the suggested strategies are solely based on routinely collected and easily accessible data (such as student absenteeism irrespective of the cause and ILI incidence) and thus they appear to be applicable in real world situations.

**Electronic supplementary material:**

The online version of this article (doi:10.1186/s12879-016-1918-z) contains supplementary material, which is available to authorized users.

## Background

Influenza disease is responsible of considerable burden in terms of morbidity and mortality worldwide every year, and concerns on its impact on the population are not fading, as the prospect of future pandemics evolving from novel influenza viruses is concrete. Schoolchildren are particularly affected by influenza [1, 2]; they tend to have higher contact rates than adults [3, 4] and consequently propagate infection in their families and in the community. This suggests that school closures can be an effective tool for public health envisaging influenza mitigation. Supporting evidence comes from the experience of the 2009 H1N1 pandemic, when a considerable drop in reported cases was registered in correspondence to regular school holidays in the US, Canada and Europe [5–8]. Unscheduled school closure was implemented locally (at the district or city level) during the pandemic and retrospective studies have shown its effectiveness [9–14]. Moreover, some countries already apply school closure policies routinely during the influenza season, generally relying on reactive closure (with schools closing due to high pupils absenteeism rates [14]) or reactive gradual closure (e.g., classes close first, then the entire school [14, 15]).

A recent modeling study investigated the effectiveness of a variety of targeted school closure measures for the United Kingdom [16]. Here we propose a more detailed, data-driven modeling approach to assess the effectiveness of school closure policies at the municipality level in Italy. In fact, since education and health are frequently under the jurisdiction of local authorities, the analysis of the impact of such measures at a local scale seems particularly appropriate. Confirming the findings in [16] by considering a different modeling approach and on a different country would greatly support the applicability of targeted school closure strategies as effective options for influenza mitigation.

## Methods

We developed an individual-based model for influenza transmission integrating data on sociodemography and time use on the Italian population, face-to-face contacts in a primary school, influenza natural history, and on the epidemiology of the 2009 influenza pandemic in Italy. The model can be seen as the combination of two layers: the population model and the infection transmission process.

### Population model

The population layer is a refined version of the model proposed in Ajelli et al. [17]. We simulate a population of 100,000 individuals (roughly corresponding to the size of an average Italian municipality) grouped in households in such a way as to match available statistics on household size, composition and age distribution of its members conditioned to its size. Individuals are also assigned, according to an age-dependent probability, to schools and workplaces, whose size matches available statistics. Students of each school are further subdivided in classes and grades, according to their age and data on class size. Moreover, at each time step of the simulation, each individual can move between different locations (namely, house, school, workplace, and the general community) on the basis of time-use data, contingent on age, employment (stratified as workers, inactive individuals, and five types of students according to the attended school level – ranging from pre-primary education to university), and day of the week (workdays, Saturdays, and Sundays), at 10-min time resolution. Moreover, time-use data allowed us also to estimate the background absenteeism of students; in particular we found that the daily fraction of students who are absent from school is 6.1 % in primary schools, 7.3 % in lower secondary schools, and 11.1 % in upper secondary schools. Further details are reported in the Additional file 1: Supplementary Material (SM).

### Infection transmission model

The infection transmission process is based on a SLAIR (Susceptible, Latent, infectious Asymptomatic, Infectious symptomatic, Removed) scheme. A susceptible individual can acquire the infection through contacts with infectious individuals (either symptomatic or asymptomatic) and become latent. A latent individual can either develop symptoms (corresponding to infectious symptomatic individuals in our scheme) or have mild/no symptoms (infectious asymptomatic individuals in our scheme). An infectious individual (either symptomatic or not) can transmit the infection to susceptible individuals belonging to their specific network of contacts. The definition of the force of infection, given a contact between susceptible and infectious individuals who are co-located in the same place at the same time, is similar to that used in [17] and accounts for contacts in households, schools, workplaces and the general community. Once no longer infectious, an asymptomatic individual becomes fully immune to the infection (removed individuals in our scheme). A symptomatic individual is initially able to transmit the infection to individuals in his whole network of contacts; however, according to evidence on influenza transmission in schools [18], the day after symptom onset, we suppose symptomatic individuals to remain at home for 3.7 days on average [19], thus reducing the network of possible contacts to household members only. Accounting for this behavior allows mimicking the absenteeism of infectious symptomatic students from schools. A detailed description of the infection transmission process is provided in the Additional file 1: SM.

### Model calibration

The model is calibrated in such a way as to produce simulations coherent with the existing evidence on the 2009 influenza pandemic. Specifically, as reference scenario, we calibrate the model to produce a reproduction number (corresponding to the average number of secondary cases generated by a primary infector) of 1.4, according to estimates specific for Italy [17, 20–22] and close to the values observed in other countries [23]. Symptomatic and asymptomatic infectious individuals are assumed to transmit with the same transmission rate. The latent period (assumed equal to the incubation period) lasts on average 1.5 days [24] and the infectious period 1.2 days, in such a way as to obtain a gamma-distributed generation time of 2.7 days [6, 18, 25]. A major determinant of the spread of the 2009 pandemic, that was evident since the beginning of the outbreak, is an age-specific susceptibility to infection [6, 25, 26]. In particular, we assume that the hazard of infection for adults (meaning individuals aged 19 years or older) given a contact is 0.2 times that of children, according to the estimate provided in [17] and obtained by using a model closely related to that employed in this study (mean 0.2, 95 % CI: 0.12–0.28). By analyzing data on face-to-face contacts in a primary school made available in [27], we found that 72.6 % of contacts are between classmates, 9.9 % between students of the same grade (but not of the same class), and 17.5 % between students of different grades or with teachers; therefore, we set different transmission rates within schools to reproduce such heterogeneous mixing patterns (see Additional file 1: SM for details). According to [28], we consider the probability of developing symptoms to be 30 %. As clearly emerged from serosurvey data (see for instance [29–31]), there were individuals already protected from infection thanks to pre-pandemic immunity; in the model we use age-specific immunity profiles as available for Italy [17, 22]. Further details on model calibration are reported in the Additional file 1: SM.

Overall, such a model calibration procedure allows the simulation of a scenario mimicking the spread of the 2009 H1N1 pandemic influenza in an Italian municipality. An extensive sensitivity analysis on model parameters is performed in order to investigate how different biological features may impact the effectiveness of different school closure policies in mitigating influenza spread.

### School closure policies

We test three different reactive school closure strategies involving all primary, lower and upper secondary schools:

1. Strategy S. If student absenteeism in a school on a certain day exceeds a threshold, the whole school will remain closed for a certain period, starting from the following day.
2. Strategy CS. If student absenteeism in a class on a certain day exceeds a threshold, that specific class will remain closed for a certain period, starting from the following day. If five or more classes of the same school appear to be simultaneously closed on the following day, the whole school will remain closed for a certain period, starting from the following day.
3. Strategy CGS. If student absenteeism in a class on a certain day exceeds a threshold, that specific class will remain closed for a certain period starting from the following day. If two or more classes of the same grade appear to be simultaneously closed on the following day, all classes of that specific grade are closed for a certain period, starting from the following day. If two or more grades of the same school appear to be simultaneously closed on the following day, the whole school will remain closed for a certain period, starting from the following day.

The evaluated strategies thus entail an increasing level of graduality in the closure: starting from the closure of the whole school (strategy S), to the closure of classes and then the whole school (strategy CS), and finally to the closure of classes, then grades and finally the whole school (strategy CGS). Strategies CS and CGS are inspired by the school closure policies currently used to mitigate the spread of seasonal influenza in Russia [15] and Japan [14], respectively. All strategies are reactive (i.e., school closure occurs as a reaction to a specific event) and are based on student absenteeism, irrespective of its cause, which is an easy-to-monitor quantity. In addition to this, we consider a trigger for the monitoring of school absenteeism based on the incidence of weekly cases in the overall population – monitoring starts as soon as the weekly incidence of new symptomatic cases reaches the reference value of 1.5 cases per 1000 individuals. We also set as reference value the length of each closure to be one week and impose that structures (e.g., classes, grades, schools) stay open for at least 1 week between consecutive closures. We perform an in-depth sensitivity analysis (consisting in about half million simulations) on the parameters regulating closure policies: namely, the trigger for the monitoring of school absenteeism, the duration of each closure, the maximum number of times that a specific structure may be closed over the course of the epidemic, and the trigger for closures, which is computed as an excess absenteeism threshold over the physiological absenteeism rate (see Additional file 1: SM).

School closure strategies entail a cost for the society (e.g., lessons lost by students, working days lost by parents to take care of children whose class or school is closed). Therefore, policy makers and response planners have to weigh the benefit of school closure policies against their costs, and their decision depends on many factors such as the severity of the epidemic and the effectiveness of the closure strategy in mitigating its spread. Similar to [16], here we measure the effectiveness of strategies in terms of reduction in attack rate and in peak week incidence and the effort that decision makers are ready to pay in terms of the “theoretical” number of (school) weeks lost, which is defined as the maximum allowed number of weeks that each school as a whole can remain closed over the entire course of the epidemic.

## Results

### Reference scenario: 2009 H1N1 influenza pandemic in the absence of interventions

**Fig. 1:**
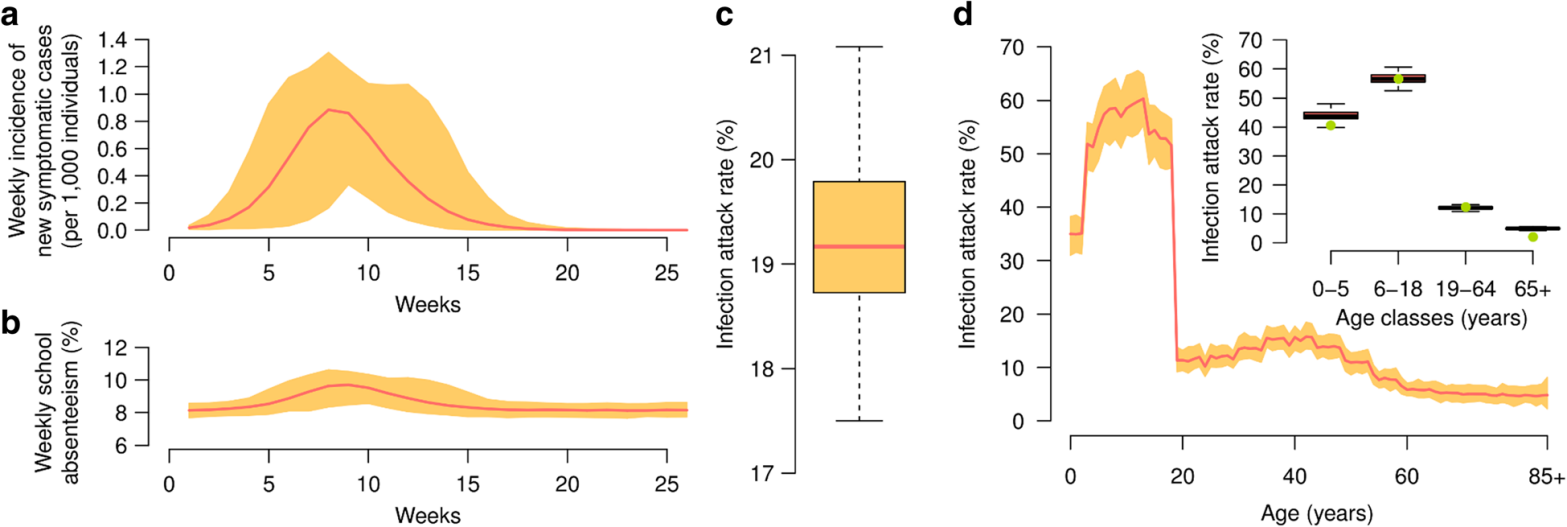
Reference scenario in the absence of interventions. All simulations shown are based on reference parameter values (reported in Sec. Methods) and initialized with 10 infectious individuals. We ran 100 stochastic realizations of the model and based our results on those simulations that caused an epidemic (i.e., more than 150 total symptomatic cases). **a** Simulated mean weekly incidence of new symptomatic cases per 1000 individuals (line) and 95 % CI (area). **b** Simulated mean weekly school absenteeism (line) and 95 % CI (area). **c** Simulated infection attack rate (median, 50 % and 95 % CI). **d** Simulated mean infection attack rate by age (line) and 95 % CI (area). The subpanel shows the distribution (median, 50 % and 95 % CI) of the simulated infection attack rate by age group (boxplots); dots represent infection attack rates (difference between post- and pre-pandemic seroprevalence) as reported in serosurvey data on the 2009 H1N1 influenza pandemic in Italy [17, 22]

In the absence of interventions, the simulated epidemics show a mean peak weekly incidence of 8.9 symptomatic cases (95 % CI: 1.6–13.0) per 10,000 individuals and an overall infection attack rate of 19.2 % (95 % CI: 17.9–20.6 %), see Fig.1a and c. In model simulations, the number of weekly cases is correlated with student absenteeism, reaching a peak of 9.6 % absent students on average on the same week when the epidemic shows its maximum incidence (Fig. 1b). Simulated infection attack rate (Fig. 1d) is highly dependent on age and compares well with serosurvey data on the 2009 H1N1 influenza pandemic in Italy [17, 22]: school-age individuals represent clearly the most affected portion of society (with 56.5 % of individuals aged 6–18 years affected), while individuals aged 65 years or older represent the less affected age group (with an infection attack rate lower than 10 %).

### Effectiveness of school closure strategies

**Fig. 2:**
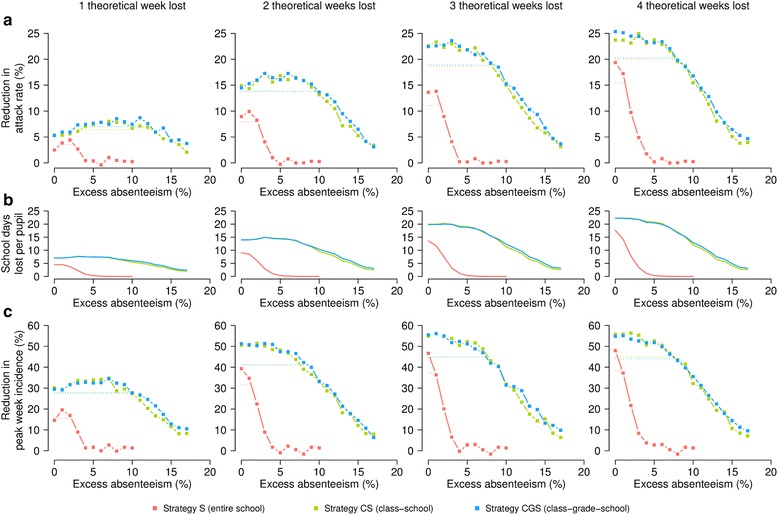
Effectiveness of default school closure strategies. All parameters involved take the reference values: generation time T_g_ = 2.7 days, effective reproduction number R_e_ = 1.4, relative susceptibility to infection of adults with respect to underaged individuals = 0.2, probability of becoming symptomatic upon infection p_I_ = 30 %, relative transmission rate of symptomatic and asymptomatic individuals = 1, trigger for the monitoring of schools = 150 new weekly symptomatic cases, length of a single closure = 1 week, minimum interval between consecutive closures = 1 week. The panels refer to school closures where the maximum number of weeks lost is one, two, three and four respectively (see *columns*). Colors refer to the three default strategies considered (see *legend*). Means are computed over 100 stochastic model realizations. **a** Reduction in attack rate for the three default strategies. *Dashed horizontal lines* represent the interval of excess absenteeism for which the obtained attack rate reduction is at least 80 % that of the maximum. **b** Effective school days lost per student for the three default strategies. **c** Reduction in peak week incidence for the three default strategies. *Dashed horizontal lines* represent the interval of excess absenteeism for which the obtained peak week incidence reduction is at least 80 % that of the maximum

Our results highlight that, independently of the number of theoretical weeks lost, the identification of the optimal excess absenteeism values triggering closures is a major determinant of policy effectiveness (see Fig. 2). Specifically, we found that closing the entire school (strategy S) when the optimal excess absenteeism threshold is adopted leads to about 4.4, 9.9, 13.8, and 19.4 % attack rate reduction for one, two, three, and four theoretical weeks lost, respectively (Fig. 2a) – an assessment of the variability of these estimates is provided in the Additional file 1: SM. Higher reductions are observed for gradual strategies (strategies CS and CGS), which are both capable of leading to reductions of about 8, 17, 23, and 25 % for one, two, three, and four theoretical weeks lost, respectively (Fig. 2a). Such a difference between the strategies is imputable to the effective number of days lost by students; in fact, as in gradual strategies also classes, and possibly grades, can remain closed without the need of closing the entire school, the actual amount of school days lost by students due to the application of strategies can be higher than the maximum number of weeks that a school can remain closed (see Fig. 2b). In particular, we found that attack rate reduction is strongly correlated with the actual number of school days lost per student (correlation 0.99, *p*-*value* < 0.0001, for all strategies), and a linear model with regression coefficient 1.12 is capable to well explain the variation (coefficient of determination 0.99, *p*-*value* < 0.0001), which means that an increase of one (effective) school day lost per student corresponds to a decrease in the attack rate of about 1.1 %. Such a pattern is consistent for the three strategies (see Additional file 1: SM).

All evaluated strategies are based on monitoring student absenteeism irrespective to the cause; however, an important distinction has to be made: strategy S is based on the monitoring of student absenteeism in the whole school, while strategies CS and CGS are based on the monitoring of absenteeism in classes, which are at least one order of magnitude smaller than schools. Clearly, the closure of single classes as a consequence of an increase in absenteeism not related to influenza yields a more acceptable burden than the closure of the entire school. On the other hand, this implies that the same percentage of absent students triggering closure corresponds to a much higher number of absent students for strategy S than for gradual strategies, suggesting that small variations in the excess absenteeism threshold would have a higher impact in strategy S than in the others. This introduces a second important aspect that has to be taken into account when considering school closure policies as intervention options: the stability of the effectiveness of the strategies with respect to the choice of the absenteeism threshold. In fact, a highly effective strategy that however requires a very precise level of absenteeism to be effective would be hardly applicable in real world situations. We consider a strategy to be stable if the range of excess absenteeism thresholds yielding at least 80 % (or 90 %) of the maximum reduction possible is wide. As clearly highlighted in Fig. 2a, strategy S has a low level of stability, while strategies CS and CGS are remarkably more stable: an excess absenteeism in the range 3.3–12.5, 0–9.5, 0–8.2, and 0–7.8 % (strategy CS) and 2.7–12.4, 0–9.9, 0–8.5, and 0–7.8 % (strategy CGS) leads to an attack rate reduction close to that obtained for the optimal value for one, two, three, and four theoretical weeks lost, respectively. These values also suggest that, in case decision makers were inclined to keep the social cost low, it would be more effective to close schools (and classes) when there is less uncertainty on the cause of student absenteeism.

All evaluated strategies are highly effective in reducing the peak weekly incidence: an increase of one (effective) school day lost per student corresponds to a decrease of the peak week incidence of about 2.6 % (see Additional file 1: SM). In particular, for one theoretical week lost, we estimate a maximum peak week incidence reduction of about 19.6 % for strategy S, and of about 34.5 % for both gradual strategies; for two or more theoretical weeks lost, the estimated maximum reduction reaches about 39–48 % for strategy S, and about 51–56 % for both gradual strategies (Fig. 2c). Again, gradual strategies show a much higher level of stability of the results with respect to the threshold of excess absenteeism than strategy S. Finally, we also found that, except for two theoretical weeks lost, the optimal threshold levels of excess absenteeism for reducing attack rate are similar to those for reducing peak incidence, highlighting that the analyzed school closure policies are highly beneficial for mitigating influenza spread in several ways. In the case of two theoretical weeks lost, the highest reduction in peak week incidence is estimated to occur when the policies are applied in the early phase of the epidemic (with all schools closing almost simultaneously), while for reducing the attack rate it would be preferable to have less uncertainty on the causes of student absenteeism by waiting to reach a higher excess absenteeism.

Finally, results suggest that the mitigation effects of strategies tend to vanish for increasingly high values of the excess absenteeism; this is due to the growing difficulty in reaching such high values of absenteeism, which results in poor (or failure in the) application of strategies.

### Sensitivity of school closure strategies to intervention parameters

**Fig. 3:**
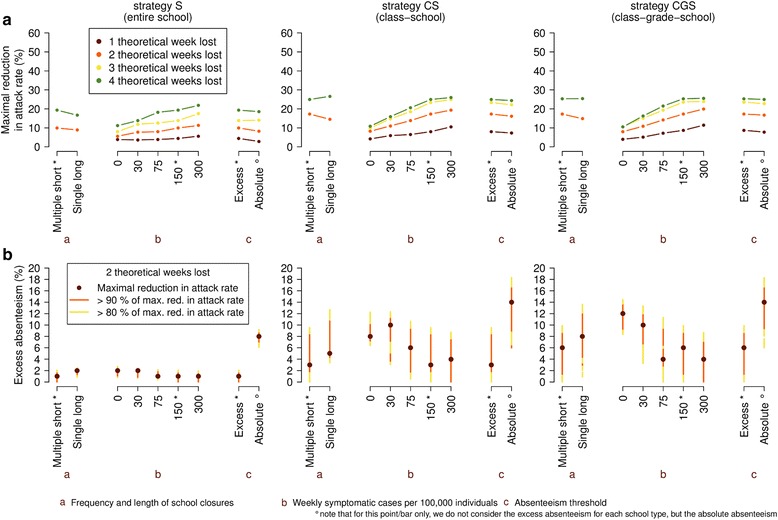
Sensitivity of school closure strategies to intervention parameters. Unless otherwise stated, the parameters involved take the reference values: generation time T_g_ = 2.7 days, effective reproduction number R_e_ = 1.4, relative susceptibility to infection of adults with respect to underaged individuals = 0.2, probability of becoming symptomatic upon infection p_I_ = 30 %, relative transmission rate of symptomatic and asymptomatic individuals = 1, trigger for the monitoring of schools = 150 new weekly symptomatic cases, length of a single closure = 1 week, minimum interval between consecutive closures = 1 week. a-c denotes the analyzed features (see Additional file 1: SM for details on absolute absenteeism). *Asterisks* on intervention parameters indicate the default values. Means are computed over 100 stochastic model realizations. **a** Maximum reduction in infection attack rate for the strategies with varied intervention parameters for one, two, three, and four theoretical weeks lost. **b** Ranges of excess absenteeism for which the attack rate reduction for the strategies with varied intervention parameters is 90 % (*red*) and 80 % (*yellow*) that of the maximum value. Only strategies yielding two theoretical weeks lost are considered. Dots denote where the maximum is attained

So far, we have shown that the effectiveness of the three strategies strongly depends on the number of theoretical weeks lost and on the threshold in student absenteeism. However, there are other parameters whose effect on the regulation of strategies needs to be investigated. In particular, in order to limit the effort required to each school (e.g., in terms of logistic organization), decision makers may be inclined to consider 2 or 4 weeks lost per student as an affordable cost, but only in form of a single closure episode. We show that, assuming a fixed number of theoretical weeks lost, results are quite stable for all strategies with respect to changes in the number of closure events (see Fig. 3A and Additional file 1: SM), provided that the proper threshold for the excess absenteeism of students is chosen (Fig. 3B). In general, we found that single closure episodes achieve better results for higher threshold levels (i.e. when there is less uncertainty on the cause of such high absenteeism rates), since longer closures are able to disrupt disease transmission more effectively.

For all strategies, results remain very stable also with respect to changes in the start of student absenteeism monitoring in schools and, consequently, in the application of school closure policies (see Fig. 3A and Additional file 1: SM). In particular, we found slightly better results when starting to monitor student absenteeism after a higher incidence (i.e., more than 75 weekly symptomatic cases per 100,000 individuals) in the total population has been observed, suggesting again that the three strategies are more effective if applied when there is low uncertainty that an epidemic is under way. Such finding is confirmed by the optimal value for the threshold on student absenteeism, which generally increases as the trigger incidence decreases (see Fig. 3B). In sum, these findings suggest that monitoring influenza-like illness (ILI) incidence - which collects information that is routinely reported to surveillance systems of health authorities - to ascertain the presence of an ongoing influenza epidemic would be sufficient as trigger for starting the monitoring of student absenteeism in schools.

Since background student absenteeism is variable by school type (i.e., larger in secondary than in primary schools), by considering excess absenteeism to determine school (and class) closure, we are assuming different absolute levels of absenteeism for each school type. Nonetheless, our findings show that this choice does not undermine the mitigation potential of the three strategies; indeed, results remains consistent if we consider a unique threshold on student absenteeism in all types of schools instead of excess absenteeism (Fig. 3A and B).

### Sensitivity of school closure strategies to biological and behavioral characteristics

**Fig. 4:**
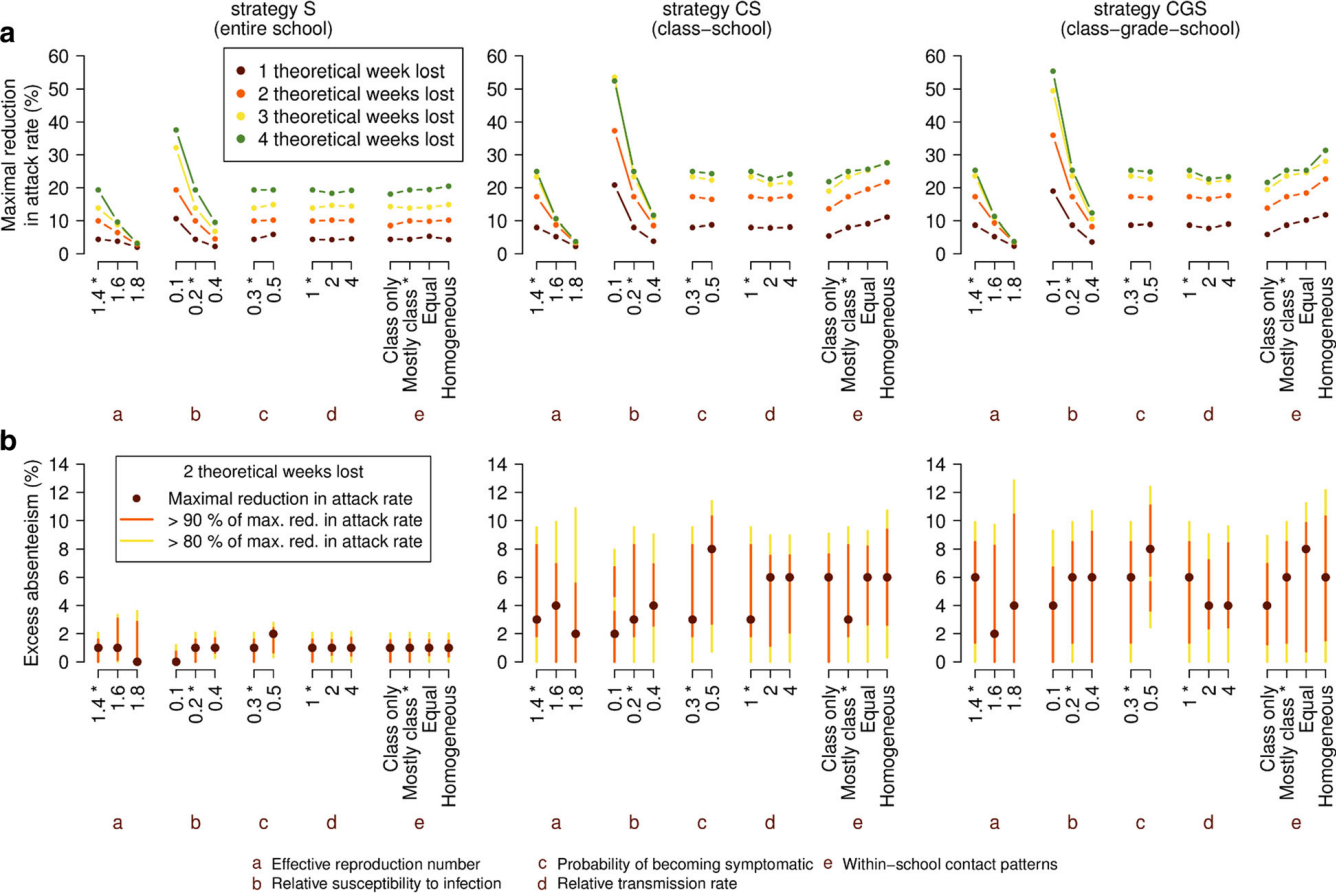
Sensitivity of school closure strategies to biological and behavioral characteristics. Unless otherwise stated, the parameters involved take the reference values: generation time T_g_ = 2.7 days, effective reproduction number R_e_ = 1.4, relative susceptibility to infection of adults with respect to underage individuals = 0.2, probability of becoming symptomatic upon infection p_I_ = 30 %, relative transmission rate of symptomatic and asymptomatic individuals = 1, trigger for the monitoring of schools = 150 new weekly symptomatic cases, length of a single closure = 1 week, minimum interval between consecutive closures = 1 week. a-e denotes the analyzed features (see Additional file 1: SM for details on within-school mixing patterns). *Asterisks* on intervention parameters indicate the default values. Means are computed over 100 stochastic model realizations. **a** Maximum reduction in infection attack rate for the strategies with alternative biological and behavioral characteristics for one, two, three, and four theoretical weeks lost. **b** Ranges of excess absenteeism for which attack rate reduction for the strategies with alternative biological and behavioral characteristics is respectively 90 % (*red*) and 80 % (*yellow*) that of the maximum value. Only strategies yielding two theoretical weeks lost are considered. *Dots* denote where the maximum is attained

Epidemic spread and effectiveness of intervention options largely depend on several factors (such as reproduction number and severity) that, unlike parameters regulating intervention strategies, are not under control of decision makers. Our findings highlight the predominant role of reproduction number and age-specific susceptibility to infection in determining the effectiveness of school closure policies. In particular, as shown in Fig. 4A, we found that, although school closure is highly effective in a 2009 pandemic scenario, for remarkably higher reproduction numbers (effective reproduction number of 1.8) none of the three strategies is capable to effectively reduce the attack rate (reductions result to be lower than 5 %). This suggests that in the case of highly transmissible influenza viruses, combined strategies relying on several interventions (e.g., vaccination as well as household quarantine) would be required to mitigate the spread of influenza pandemic/epidemic [32–34]. On the other hand, by fixing the value of the reproduction number and varying the age-specific susceptibility to infection, we found that the more susceptible to infection children are with respect to adults, the more effective school closure policies become. In particular, in a scenario where children are twice as susceptible to infection as in the 2009 pandemic, at three theoretical weeks lost strategies CS and CGS entail an attack rate reduction of about 50 % (Fig. 4A) and a peak week incidence reduction larger than 50 % (see Additional file 1: SM). Again, gradual strategies CS and CGS result to be more effective than strategy S and remarkably less sensitive to the excess absenteeism threshold, consequently allowing for a more manageable application in real-world situations (see Fig. 4A and B).

The effectiveness of school closure policies appears to be much less affected by changes in the symptomatic probability and by variations in the transmission rate of symptomatic infectious individuals with respect to asymptomatic ones: comparable attack rate reductions are observed for all three strategies (Fig. 4A). Finally, we found that gradual strategies are more effective also if contacts between students at school are homogeneous, suggesting that the trigger on classes works better than that on the whole school (see Fig. 4A). In fact, more homogenous contacts within school entail a larger dispersion of the infection inside the school and thus a larger number of classes to be closed simultaneously by the strategies and, in turn, a more immediate closure of the entire school as a reaction to influenza spread (see Additional file 1: SM); on the other hand, closures of single classes (possibly asynchronous) prevail if transmission is related only to contacts within the class, allowing the infection to circulate in non-closed classes and hence propagate to the general population. For the two intermediate scenarios, one considering the default within-school contact patterns resulting from data analysis [18, 27] and one where classes, grades and the overall school have the same weight in infection transmission, we found that the different school closure policies yield very similar effectiveness. Strategy S is generally not sensitive to variations in within-school contact patterns (Fig. 4A).

### Comparison with targeted school closure strategies for the UK

**Fig. 5:**
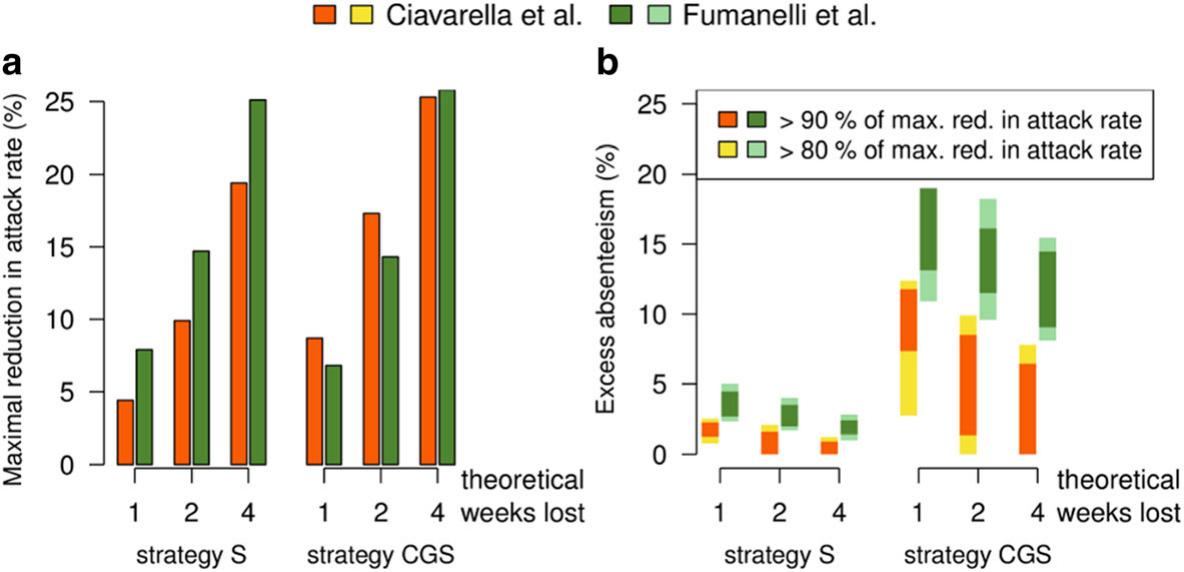
Comparison with school closure strategies for the UK. **a** Maximal attack rate reduction for one, two and four theoretical weeks lost for strategies S and CGS. **b** Ranges of excess absenteeism for which the attack rate reduction for the strategies is 90 % (*dark colors*) and 80 % (*light colors*) that of the maximum value. Data for the UK are taken from Fumanelli et al. [16]

We compare our results with those presented in Fumanelli et al. [16] relative to the UK. The two studies differ in three substantial modeling aspects: first, our model accounts for data on time use and on face-to-face contacts in a primary school (see Section Methods), while in [16] a simpler model based on a static network of contacts between individuals was employed; second, we investigate the effectiveness of school closure strategies at the level of a single municipality, while [16] was focused on the national scale; third, the analyzed country is different. Clearly, considering two different countries requires resorting to nation-specific data, for instance on the socio-demographic characteristics of the population and thus on contact pattern, background absenteeism, school size and structure. In addition, although both works consider the 2009 H1N1 pandemic as the reference scenario, the different characteristics of the two countries give origin to two different epidemics even in the absence of interventions (e.g., R_0_ = 1.4 here, R_0_ = 1.5 in [16]). However, we are considering the same pandemic scenario and strategies S and CGS are implemented the same way, thus a comparison between the two is possible. We find that attack rate reductions are comparable in case of strategy S and even more similar for strategy CGS (Fig. 5a); moreover, both models show that strategy CGS is much more stable than strategy S with respect to the choice of the absenteeism threshold (Fig. 5b), suggesting that the gradual closure approach may be preferable in real-world situations. In both models the optimal excess absenteeism threshold decreases as the number of weeks of closure increases (Fig. 5b); its value differs between the two models probably as a consequence of the different average level of background absenteeism, average school and class size of the two countries.

## Discussion

Our simulation results suggest that reactive school closure policies based on student absenteeism can be highly effective in mitigating influenza spread in the case of pandemics/epidemics characterized by features (e.g., reproduction number and age-specific susceptibility to infection) comparable to the 2009 H1N1 pandemic. In particular, we estimate that gradual school closure strategies (i.e., strategies where classes close first) could lead to an attack rate reduction up to 20–25 % and to a peak week incidence reduction up to 50–55 %, at the cost of about three school weeks lost per student. Gradual strategies result to be quite stable to variations in the start of policy application – they would only require to be applied when the epidemic is ongoing (which can be ascertained, e.g., by monitoring ILI incidence) – and to the threshold on student absenteeism that triggers class (and school) closure – optimal results are found by considering excess absenteeism in the range 2–9 %. On the other hand, a closure policy accounting for the closure of the whole school after a certain threshold on absent students in the whole school is reached appears to be harder to apply in practice; in fact, in order to obtain reductions in attack rate and peak week incidence (up to 13.8 and 46.6 %, respectively, at the cost of three school weeks lost per student) similar to those obtained with gradual closures, it is required to consider a very precise student absenteeism threshold, which might be hardly identifiable in practice. Moreover, all tested strategies are based on monitoring student absenteeism to trigger class (and school) closure, which is an easy-to-monitor quantity; in fact, a proper identification of influenza cases in schools would not be feasible in real-world situations. In sum, our findings suggest that gradual strategies may be applicable in real-world situations and be highly effective to mitigate the influenza spread in a scenario close to that of the 2009 H1N1 pandemic.

In the case of an influenza epidemic/pandemic showing considerably different characteristics (e.g., severity and reproduction number) with respect to those of the 2009 H1N1 pandemic, the effectiveness of school closure policies changes remarkably. In particular, obtained results show that the efficacy of school closure policies is not particularly sensitive to variations in the probability of developing clinical symptoms and in the relative infectiousness of symptomatic and asymptomatic individuals; on the other hand, results strongly depend on the reproduction number, age-specific susceptibility to infection, and contact patterns between students at school. Our findings strongly suggest that reducing the uncertainty in the estimates of reproduction number and age-specific susceptibility to infection is essential in order to support policy makers in the design of optimal response plans. In particular, we found that in case children were extremely more susceptible than adults, influenza spread could be mitigated by school closure policies alone (up to 50 % reduction both in attack rate and peak week incidence), while in the case of a highly transmissible infection (effective reproduction number around 1.8), school closure policies alone reduce the attack rate by about 5 % at most, and should thus be combined with other strategies in order to effectively reduce the epidemic burden [32–34]. However, our findings also suggest that school closure policies have a beneficial effect, at least to some extent, independently of the value of the reproduction number and lead to a noticeable reduction of peak week incidence even for large values of the reproduction number.

Other crucial determinants of the effectiveness of the considered strategies could have been investigated. For instance, it would be of great use to know whether school closure policies could effectively be employed to delay the epidemic spread of a novel influenza strain in order to gain time for the development and administration of an appropriate vaccine to the population [31, 32]. However, our model is not designed to perform such analyses since it lacks a spatial component (which significantly impacts the timing of an epidemic spread [34–36]) and neglects importation of cases during the course of the epidemic. This was indeed a deliberate modeling choice made in favor of a highly detailed model informed on contact patterns at the highest available resolution (i.e. time-use data at a 10-min timescale along with within-school contact patterns). Through the use of these data, the model reproduces features such as background student absenteeism and heterogeneous mixing between students at school, which have emerged to be key features necessary for a proper evaluation of school closure policies. This was done at the cost of a significant increase in computation time that ultimately posed a constraint on the simulated population's size and spatial distribution. Furthermore, the model does not account for other factors such as absolute humidity that has been shown to possibly alter influenza spread [37]. Indeed, we reasoned that the small spatial scale and the relatively short duration of the epidemic on a municipality level (about 3 months) would naturally hinder the occurrence of high climatic variability and thus decided to omit this aspect from our analysis. Finally, data on the behavior of students forced not to attend school because of closure policies would be of primary interest to allow for a more accurate assessment of school closure strategies. Unfortunately, such data has yet to be collected and we dare hope that specific studies performed, for instance, in countries like Russia and Japan that are currently employing school closure strategies for seasonal influenza, will be planned soon. Moreover, especially in the case of a sever pandemic, parents might voluntarily decide to hold their children out of school, possibly altering the results of our analysis: this highlights the need to account for spontaneous behavioral changes in future model-based evaluations of intervention strategies [21, 35–39].

## Conclusions

Our results are broadly consistent with those in Fumanelli et al. [16], which however referred to the UK and were obtained by using a different methodological approach. Only small variations are observed, which are mainly attributable to differences in the school system (such as school/class size, school composition) and sociodemographic features (such as fraction of students in the population, background absenteeism). This highlights the need of designing country-specific interventions. However, the methodological framework we have proposed here is based on routinely collected and easily accessible data, and thus the proposed school closure strategies can be tuned for other countries. In conclusion, our results highlight that reactive gradual school closure policies are applicable in real-world situations with beneficial effects in mitigating influenza spread, conditioned on the effort that decision makers are willing to afford.

## Additional file

Additional file 1: Supplementary Material. Text describing methods in detail and additional results. (PDF 461 kb)

## Abbreviations

ILI: Influenza-like illness
SM: Supplementary material
Strategy CGS: Strategy consisting in the closure of classes, then grades and finally the whole school
Strategy CS: Strategy consisting in the closure of classes and then the whole school
Strategy S: Strategy consisting in the closure of the whole school
