# Supplementary material for: School closure policies at municipality level for mitigating influenza spread: a model-based evaluation
Source: BMC Infect Dis. 2016 Oct 18;16:576. doi: 10.1186/s12879-016-1918-z (PMC5070162; doi:10.1186/s12879-016-1918-z)
Supplement: Supplementary file 1 — Supplementary Material. Text describing methods in detail and additional results. (PDF 461 kb) [file 12879_2016_1918_MOESM1_ESM.pdf]

# Evaluating school closure policies at the municipality level for mitigating influenza spread

Constanze Ciavarella[1], Laura Fumanelli[1] , Stefano Merler[1], Ciro Cattuto[2], Marco Ajelli[1]

[1] Bruno Kessler Foundation, Trento, Italy
[2] ISI Foundation, Turin, Italy

## Supplementary Material

## Contents

**1 Detailed methods** **2**
1.1 Socio-demographic model . . . . . . . . . . 2
1.2 Time-use data . . . . . . . . . . 2
1.3 Dynamic allocation of individuals . . . . . . . . . . 3
1.4 Estimating contact patterns within schools . . . . . . . . . . 4
1.5 Transmission model . . . . . . . . . . 4
1.6 Model calibration . . . . . . . . . . 6

**2 Additional results** **6**
2.1 Correlation between school closure effectiveness and effective number of school days lost 7
2.2 Variability on the effectiveness of school closure strategies . . . . . . . . . . 7
2.3 Sensitivity analysis on school closure parameters . . . . . . . . . . 10
2.3.1 Frequency and length of closures . . . . . . . . . . 11
2.3.2 Trigger for the monitoring of schools . . . . . . . . . . 12
2.3.3 Absenteeism threshold . . . . . . . . . . 13
2.4 Sensitivity analysis on epidemiological and behavioral characteristics . . . . . . . . . . 14
2.4.1 Effective reproduction number . . . . . . . . . . 16
2.4.2 Relative susceptibility to infection . . . . . . . . . . 17
2.4.3 Probability of developing symptoms . . . . . . . . . . 18
2.4.4 Relative transmission rate of symptomatic individuals with respect to asymptomatic individuals . . . . . . . . . . 19
2.4.5 Within-school contact patterns . . . . . . . . . . 20

**Bibliography** **21**

# 1 Detailed methods

## 1.1 Socio-demographic model

The socio-demographic structure of the model population is the same used in [1], which is based on high resolution Italian-specific data on age structure, household types and sizes, age-specific employment rates, schools and industry.

Differently from [1], here the modeling of schools was refined in order to allow the implementation of targeted intervention strategies: underaged individuals associated to primary or secondary schools (subsequently referred to as 'pupils') are allocated to grades of same-aged and assigned to classes. The observed mean class size is 19 pupils in primary schools [2] and 22 in lower and upper secondary schools [2, 3]. The simulated class size shows little variability and well reproduces the observed data (see Figure S1A). The model also accounts for the observed distribution of school size by school level [4, 5] (see Figure S1B).

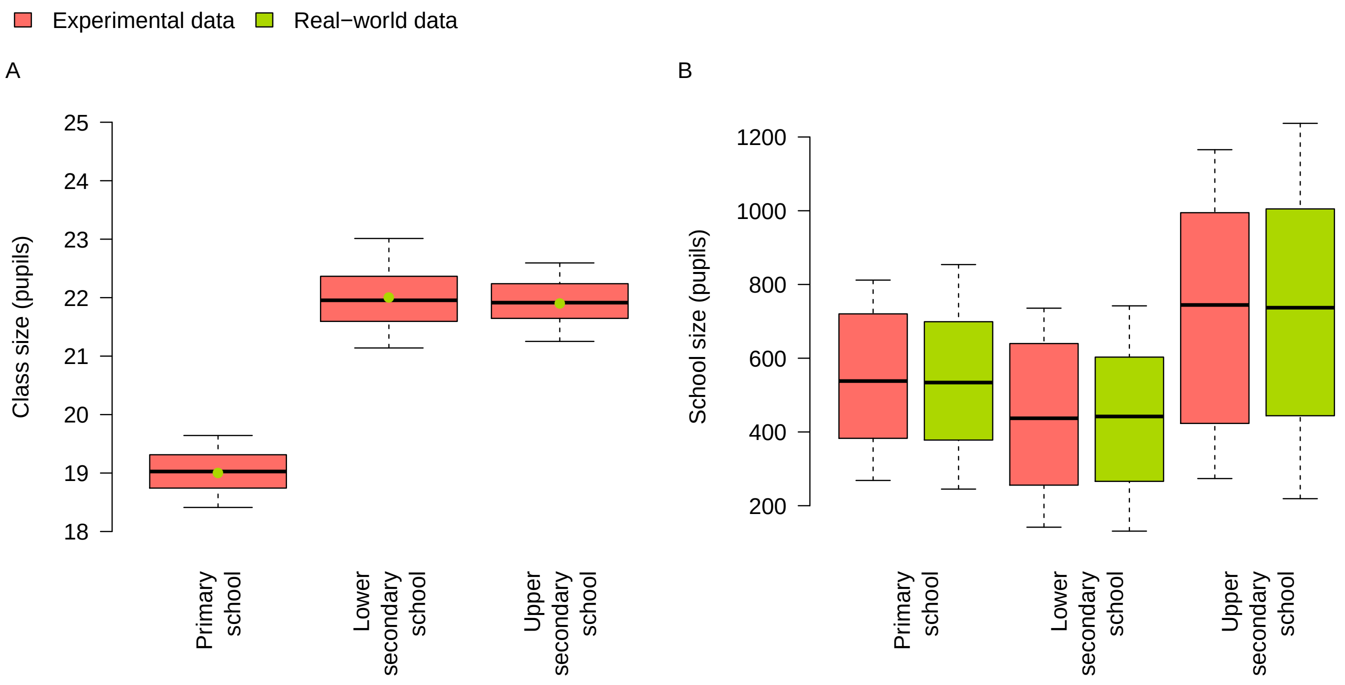


Figure S1: **Class and school size.** **A** Distribution (median, 50% CI and 95% CI) of the estimated class size (red boxes) and observed mean [2, 3] of the class size (green dots) for each considered school type (primary, lower secondary and upper secondary school). **B** Distribution (median, 50% CI and 95% CI) of the estimated school size (red) and of the observed school size (green) [4, 5] for each considered school type (primary, lower secondary and upper secondary schools).

## 1.2 Time-use data

Italian time-use data [6] provide information on the routine of 55, 773 individuals during the day and on the time spent in different social contexts. These data are supplied at a 10-minute time resolution and are stratified by age, employment and day of the week (we distinguish between working days, Saturdays and Sundays). The analysis of time-use data enables to dynamically associate individuals either to one specific location (i.e. their own household, their own school, their own workplace) or to the general community, at any given time step of the simulation and according to the simulated

day of the week. Contacts in the general community are defined to be all contacts not occurring at home, school, or workplace; for instance, the general community accounts for contacts occurring on public transportation, restaurants, shops, etc. Accounting for the time spent by individuals of different ages and employment types in different social contexts allows us to mimic the complex heterogeneous mixing of individuals within the population. To include this information, time-use data were processed to compute the probability of being located at home, school, work or general community depending on age, employment type, time of the day and day of the week. The model follows cyclically a pattern of five simulated days that employ the probability derived from time-use data referring to working days, and two simulated days referring to the time-use data of Saturdays and Sundays respectively.

By differentiating working days from weekend days, regular school closures over weekends can be explicitly modeled. In Italy each school administration autonomously decides whether pupils attend lessons on Saturdays. However, as data on the fraction of schools that are closed on Saturdays are not available, we assume in model simulations that all schools remain closed on Saturdays.

## 1.3 Dynamic allocation of individuals

In order to simulate the movements of the different members of society we resort to dynamic allocation of individuals at each time step. We illustrate the underlying algorithm with the following example. Consider unemployed individuals belonging to the age class 61-80, and let $N$ be their total number in the simulation. We indicate with $N_H(t)$ and $N_C(t)$ the number of simulated unemployed individuals aged 61-80 at time $t$ that are at home and in the general community respectively and with $p_H(t)$ and $p_C(t)$ their respective fractions; hence $p_H(t) + p_C(t) = 1$.

Let now $i$ be a simulated unemployed individual in age class 61-80. At the beginning of the simulation (midnight of the first simulated Monday), the probability for $i$ of being at home is $p_H(0)$ and that of being in the general community is $p_C(0)$. Thus, the probability distribution for $i$ to be at home or in the general community is $B(p_H(0))$, $B$ being the Bernoulli distribution and success meaning that $i$ is allocated at home. As a consequence, the average number of simulated unemployed individuals aged 61-80 at home at time $t = 0$ is $N_H(0) = p_H(0)N$. The same holds for the average number of those located in the general community.

The location of $i$ at the following time step $t = 1$ (corresponding to a 10-minute step) is determined as follows:

- If at time $t = 0$ individual $i$ was located at home, then
  - if $p_H(1) > p_H(0)$, he remains at home,
  - if $p_H(1) \leq p_H(0)$, he remains at home with probability $p_H(1)/p_H(0)$ and moves to the general community with probability $1 - p_H(1)/p_H(0)$ (employing the Bernoulli distribution).
- If at time $t = 0$ individual $i$ was located in the general community, then
  - if $p_C(1) > p_C(0)$, he remains in the general community,
  - if $p_C(1) \leq p_C(0)$, he remains in the general community with probability $p_C(1)/p_C(0)$ and goes home with probability $1 - p_C(1)/p_C(0)$ (employing the Bernoulli distribution).

As a consequence, we have that the average number of simulated unemployed individuals aged 61-80 who are at home at time $t = 1$ is

$$N_H(1) = N_H(0) + N_C(0)\left(1 - \frac{p_C(1)}{p_C(0)}\right) = N - N_C(0)\frac{p_C(1)}{p_C(0)} = N - p_C(1)N = p_H(1)N$$

if $p_H(1) > p_H(0)$ (i.e. $p_C(1) \leq p_C(0)$), and

$$N_H(1) = N_H(0)\frac{p_H(1)}{p_H(0)} = p_H(1)N$$

if $p_H(1) \leq p_H(0)$ (i.e. $p_C(1) > p_C(0)$). By induction we have

$$N = \frac{N_H(t)}{p_H(t)} = \frac{N_C(t)}{p_C(t)} \quad \text{for all } t.$$

A similar procedure is used to dynamically locate all simulated individuals, with the only difference that those who are not unemployed can be located in three different places: household, general community, school/workplace. This algorithm has been validated against the original time-use data in [1] and induces plausible behavior in the simulated individuals.

## 1.4 Estimating contact patterns within schools

In order to model transmission in schools we have to take into account the within-school contact patterns: for this aim we used data collected through proximity sensors in a French primary school [7] and consisting of a list of contacts between individuals belonging to that school during the course of two school days. Close-range interactions suitable for transmission of the influenza virus were measured between class mates, between pupils of the same grade but belonging to different classes, and across grades (including contacts with teachers, but not those among teachers). From this information we estimated the contact rate of pupils in schools as the fraction of time each pupil spends with the members of the different groups: specifically, we found that 72.6% of a pupil's contacts while in school occur with pupils of its own class, 9.9% with pupils belonging to the same grade but not the same class, and the remaining 17.5% with pupils of other grades or teachers.

## 1.5 Transmission model

Influenza transmission is modeled by coupling a SLAIR (Susceptible - Latent - infectious Asymptomatic - Infectious symptomatic - Removed) scheme with the sociodemographic model described so far. Individuals who are susceptible to the disease can get infected only through appropriate contacts with infectious individuals who are sharing the same place at the same time. Mixing between individuals sharing the same location at the same time is assumed to be homogeneous, except for pupils of primary and secondary schools where we consider a more detailed heterogeneous mixing (see Section 1.4). Susceptible individuals can acquire infection through contacts with infectious individuals (both symptomatic and asymptomatic) and become latent. After 1.5 days on average [8, 9], latent individuals can either develop symptoms (infectious symptomatic individuals in our scheme) or have mild/no symptoms (infectious asymptomatic individuals in our scheme). Infectious individuals (both symptomatic and asymptomatic) can transmit the infection to all susceptible individuals belonging to their specific network of contacts. Asymptomatic individuals become fully immune to infection (removed individuals in our scheme) after 1.2 days on average - the resulting generation time is 2.7 days [9, 10, 11, 12]. Symptomatic individuals stay at home for 3.7 days on average [13] since the day they first show symptoms (that we assume to occur simultaneously to infectiousness). Their average infectious period is 1.2 days, as for asymptomatic individuals.

The definition of the force of infection is location-specific:

$$\lambda_i(t, a_i) = r(a_i)\, \lambda^{loc(i,t)}(t),$$

where $loc(i,t)$ is the location of individual $i$ at time $t$, $a_i$ the age, and $r(a_i)$ the age-specific susceptibility to infection (see main text and Section 2.4.2).

In particular, we have

$$\lambda^H(t) = \beta\,\frac{A_H(t) + \theta I_H(t)}{N_H - 1} \quad \text{for households;}$$

$$\lambda^P(t) = \beta\,\frac{A_P(t) + \theta I_P(t)}{N_P} \quad \text{for workplaces, kindergartens and universities;}$$

$$\lambda^R(t) = \beta\,\frac{A_{tot}(t) + \theta I_R(t)}{N_{tot}} \quad \text{for the general community;}$$

$$\lambda^S(t) = \beta\,\frac{A_S(t) + \theta I_S(t)}{N_S} \quad \text{for teachers of primary and secondary schools;}$$

$$\lambda^S(t) = \beta\left(w_c\frac{A_{c(i)}(t) + \theta I_{c(i)}(t)}{N_{c(i)}} + w_g\frac{A_{g(i)}(t) + \theta I_{g(i)}(t)}{N_{g(i)}} + w_s\frac{A_{s(i)}(t) + \theta I_{s(i)}(t)}{N_{s(i)}}\right) \quad \text{for pupils of primary and secondary schools.}$$

Here $I_{loc(i,t)}(t)$ is the number of symptomatic infectious individuals in $loc(i,t)$ at time $t$, $A_{loc(i,t)}(t)$ is the number of asymptomatic infectious individuals in $loc(i,t)$ at time $t$, $N_{loc(i,t)}$ is the total number of individuals assigned to that place (not only the ones present at the specific time step $t$). $\beta$ represents the transmission rate for asymptomatic individuals and $\theta$ a scaling factor determining the relative infectiousness of symptomatic infective individuals with respect to asymptomatic ones (we assume $\theta = 1$ as baseline scenario). In addition, $N_{tot}$ denotes all the individuals in the simulation (i.e. 100,000 individuals).

| **Biological parameter** | **Value** | **Reference** | **Alternative values** |
|---|---|---|---|
| Latent period ($T_l$; days) | 1.5 | [8, 9] | – |
| Generation time ($T_g$; days) | 2.7 | [9, 10, 11, 12] | – |
| Effective reproduction number ($\mathcal{R}_e$) | 1.4 | [1] | 1.6, 1.8 |
| Relative susceptibility to infection of adults (18+ years old) with respect to underaged individuals | 0.2 | [1] | 0.1, 0.4 |
| Probability of becoming symptomatic upon infection ($p_I$; %) | 30 | [16] | 50 |
| Within-school contact patterns (class, grade, school; %) | 72.6, 9.9, 17.5 | estimated⋆ | class only (100%, 0%, 0%) equal (33%, 33%, 33%) homogeneous (entire school) |
| Relative transmission rate of symptomatic individuals with respect to asymptomatic individuals | 1 | assumed | 2, 4 |

⋆see Section 1.4.

Table S1: **Biological parameters used in model simulations.** Reference values and values used in the sensitivity analysis (Sections 2.3 and 2.4).

Modeling of epidemic transmission to pupils in primary and secondary schools is more detailed and heterogeneous: for any pupil $i$ of a school, the force of infection is computed as a weighted sum of the forces of infection relative to his/her class, the grade without his/her class and the school without the entire grade. The weights are given by the contact rates reported in Section 1.4 ($w_c = 0.726$, $w_g = 0.099$, $w_s = 0.175$).

### 1.6 Model calibration

The value of the effective reproduction number for the 2009 influenza pandemic in Italy has been estimated to be 1.39 (95% CI: 1.23 – 1.59) [1]. We calibrated the transmission rate $\beta$, which is the unique free parameter of the model, in such a way that the median value of the effective reproduction number $\mathcal{R}_e$ for simulated epidemics was 1.40. All other parameters are taken from the literature as reported in Table S1. In model simulations $R_e$ is computed as $R_e = (1 + r\,T_l)(1 + r\,T_i)$ [14], where $T_l$ and $T_i$ are the average length of the latent and of the infectious period, respectively, and $r$ is the rate of exponential growth of the number of weekly new symptomatic cases.

## 2 Additional results

The following analyses on the effectiveness of school closure strategies are carried out by comparison to the baseline. Thus 'reductions' are intended as reductions with respect to the baseline. The term *default strategies* will denote strategies where the reference parameter values (see Tables S1 and S2) are assumed.

| Intervention parameters | Reference values | Alternative values |
|---|---|---|
| Length of a single closure (weeks) | 1 | 2, 4 |
| Number of allowed closure repetitions (theoretical weeks lost) | 1,2,3,4 | – |
| Minimum interval between consecutive closures (weeks) | 1 | – |
| Trigger for the monitoring of schools (new weekly symptomatic cases per 100,000 individuals) | 150 | 0, 30, 75, 300 |
| Absenteeism threshold type | excess | absolute |

Table S2: **Intervention parameters used in model simulations.** Reference values and values used in the sensitivity analysis.

## 2.1 Correlation between school closure effectiveness and effective number of school days lost

A strong correlation (0.99, *p-value*$<$ 0.0001, for all strategies) exists between the effective number of school days lost per pupil and the performance of the default strategies in terms of both attack rate (Figure S2A) and peak week incidence reduction (Figure S2B). For any default strategy, an increase of one (effective) school day lost per pupil corresponds to a decrease of about 1.1% in attack rate and 2.6% in peak week incidence.

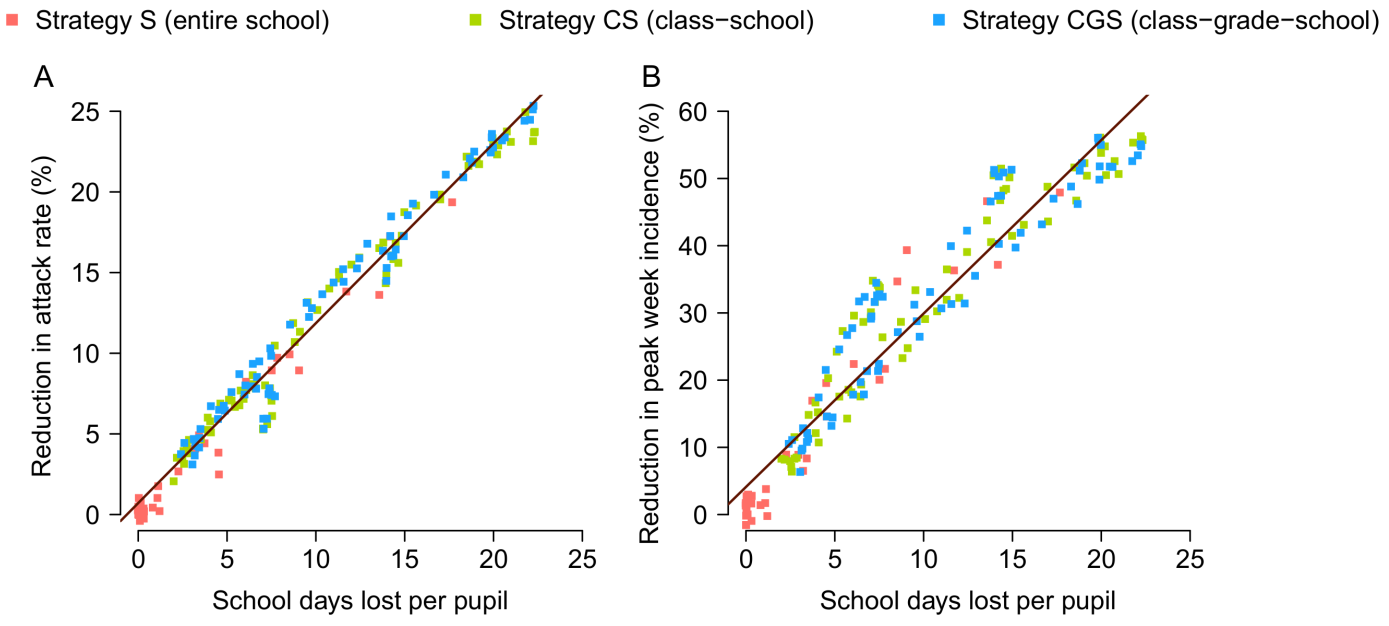


Figure S2: **School days lost per pupil with the default strategiess.** All parameters involved take the reference values reported in Tables S1and S2. **A** Correlation of attack rate reduction with the effective number of school days lost per pupil. Regression coefficient: 1.12. **B** Correlation of peak week incidence reduction with the effective number of school days lost per pupil. Regression coefficient: 2.58.

## 2.2 Variability on the effectiveness of school closure strategies

The difference between the mean attack rates for the scenario without intervention and for the best default strategies (see Fig. 2 of the main text and Fig. S3) is significant for any closure type and number of theoretical weeks lost according to unpaired t-test (p-value$<$ 0.0001). The same applies for results concerning peak week incidence. The variability of the distributions of attack rate and peak week incidence is displayed in Fig. S3.

The proportional reduction in attack rate is defined as $1 - AR_{SC}/AR_{NI}$, with $AR_{SC}$ and $AR_{NI}$ denoting the population attack rate when school closure strategies are in place or not, respectively. The standard deviation of this indicator can be computed by using standard equations for calculating propagation of uncertainty for non-linear combinations of random variables, namely:

$$\sqrt{\left(\frac{\partial f}{\partial x}\right)^2 \sigma^2(x) + \left(\frac{\partial f}{\partial y}\right)^2 \sigma^2(y)},$$

where $x = AR_{SC}$, $y = AR_{NI}$, $f(x, y) = 1 - x/y$, $\sigma$ denotes the standard deviation, and partial derivatives are evaluated at the mean value of all components $x$ and $y$.

The same equation is used for computing the standard deviation on peak week incidence. The obtained variabilities in the reduction of attack rate and peak week incidence for the best default strategies are shown in Fig. S4.

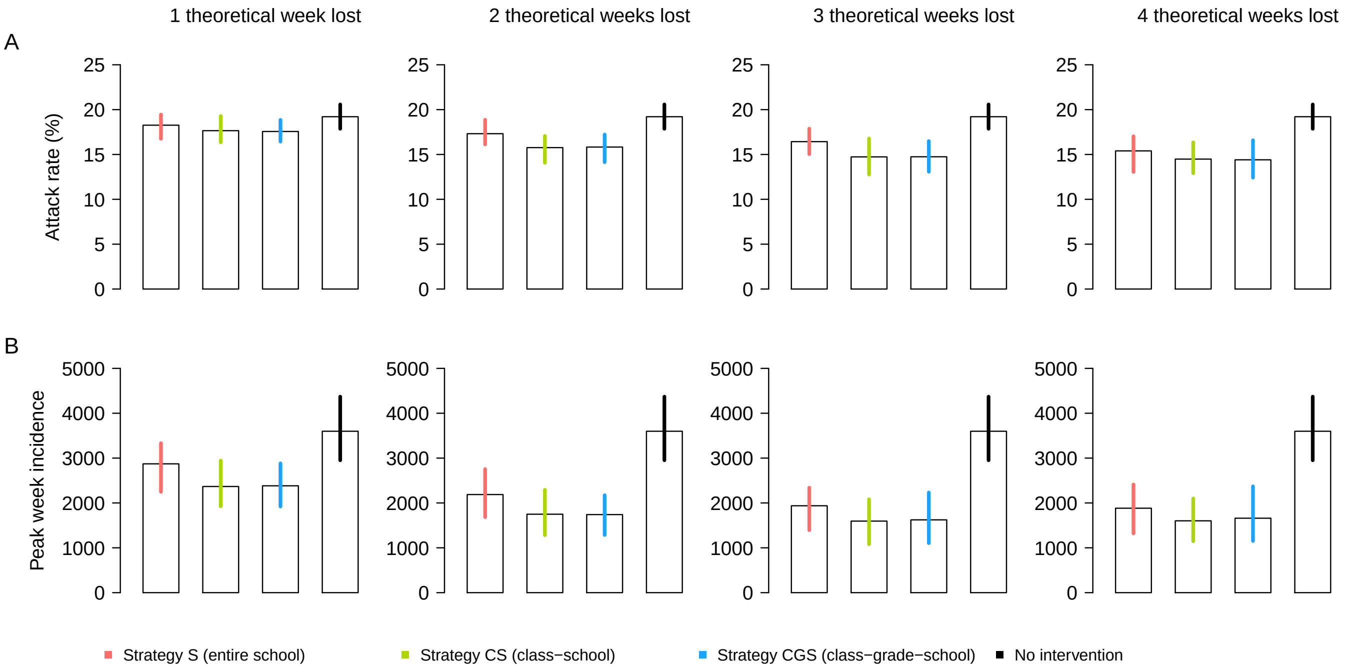


Figure S3: **Variability on attack rates and peak week incidence.** All parameters involved take the reference values reported in Tables S1and S2. **A** Attack rate (mean and 95%CI) for the simulations leading to the largest attack rate reductions for the three strategies and for the no intervention scenario. Absenteeism thresholds used can be found in Fig. 2 of the main text. **B** As A, but for peak week incidence.

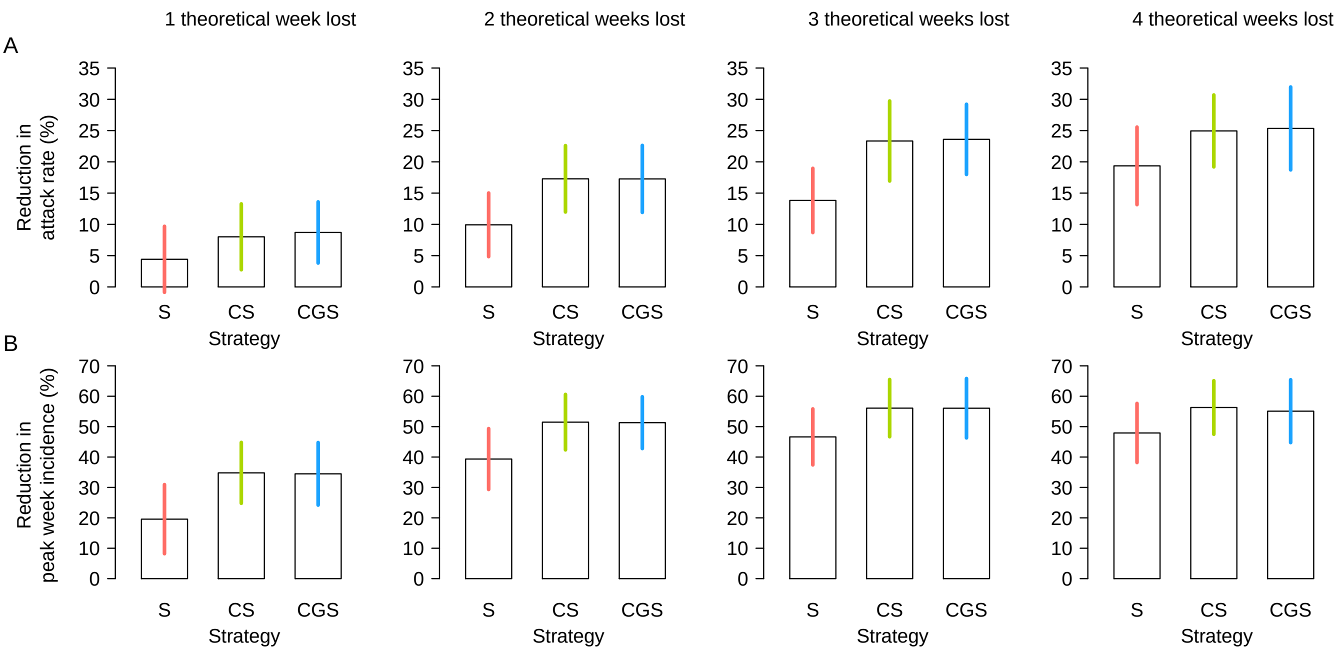


Figure S4: **Variability on the reduction of attack rate reduction and peak week incidence.** **A** Attack rate reduction (mean and 2 standard deviations) for the simulations reported in Fig. S3A. **B** Peak week incidence reduction (mean and 2 standard deviations) for the simulations reported in Fig. S3B.

## 2.3 Sensitivity analysis on school closure parameters

We investigate alternative values for the parameters regulating school closure policies (see Table S2). Three main aspects may be varied:

1. the length and frequency of closures;
2. the number of new weekly symptomatic cases that triggers the monitoring of schools;
3. the type of absenteeism threshold.

The overall impact of the variation of such characteristics on peak week incidence reduction is represented in Figure S5 (attack rate reduction is shown in the main text).

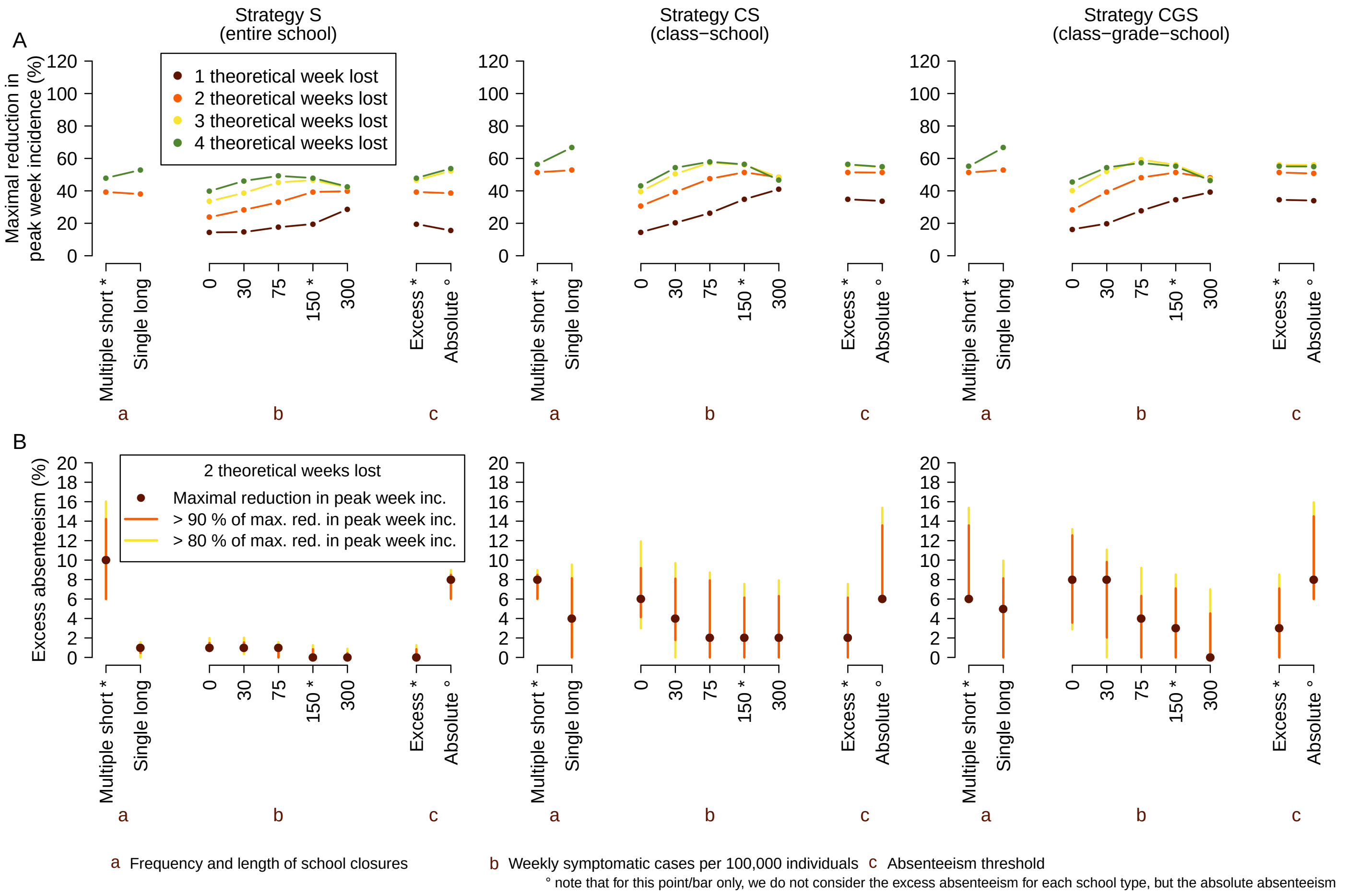


Figure S5: **Sensitivity of peak week incidence to intervention parameters.** Unless otherwise stated, parameters take the reference values reported in Table S1. a-c denote the analyzed features. Asterisks indicate the default values. Means are computed over 100 stochastic realizations. **A** Maximal peak week incidence reduction for the strategies with varied intervention parameters for 1, 2, 3, and 4 theoretical weeks lost. **B** Ranges of excess absenteeism for which peak week incidence reduction for the strategies with varied intervention parameters is at least 90% (red) or 80% (yellow) of the maximal value. Only strategies yielding 2 theoretical weeks lost are considered. Dots indicate where the maximum is attained.

### 2.3.1 Frequency and length of closures

Variations in the length of the single closure events and their frequency have little impact on the outcome of the intervention (Figure S6): in fact, the range of attack rate reduction covered by a single long closure is essentially the same as that obtained with multiple closures of one week each.

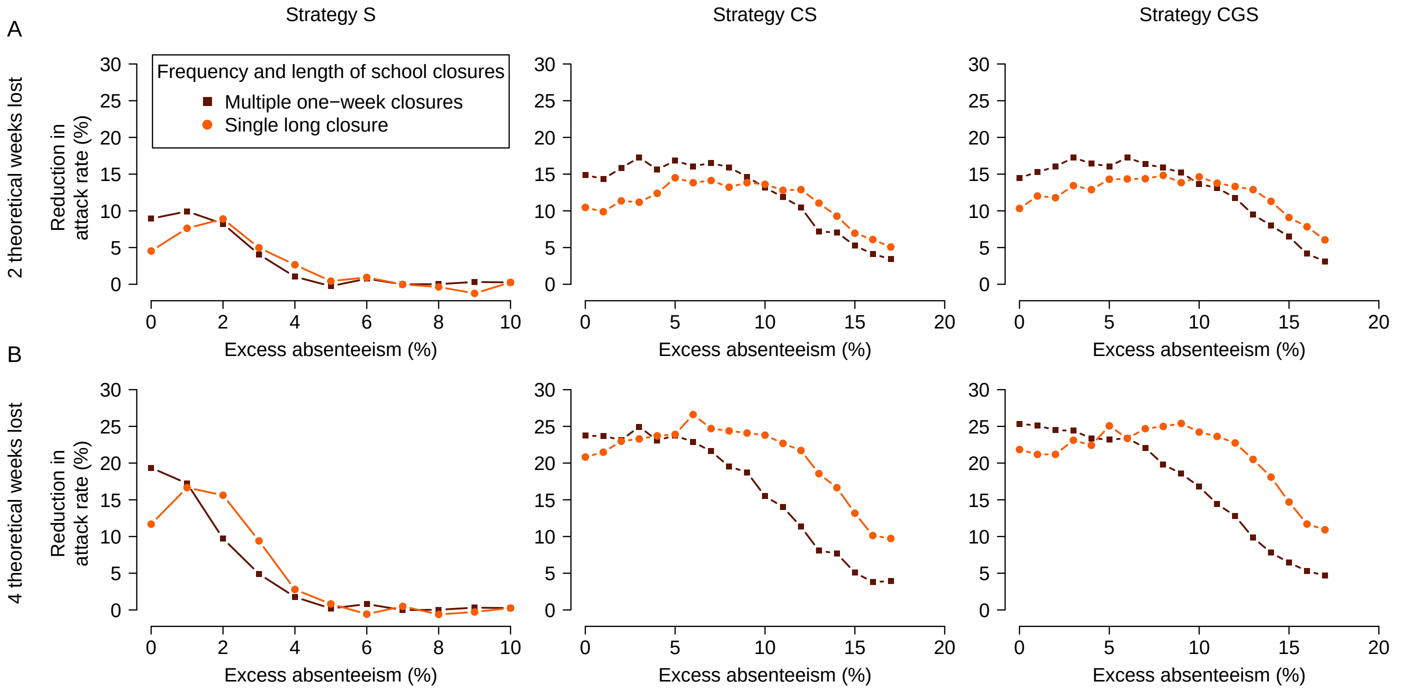


Figure S6: **Attack rate reduction for different length and frequency of closures.** All remaining parameters take the reference values reported in Table S1. **A** Effectiveness for two theoretical weeks lost. Brown: two closures of one week each, separated by one-week long intervals; red: one single closure lasting two weeks. **B** Effectiveness for four theoretical weeks lost. Brown: four closures of one week each, separated by one-week long intervals; red: one single closure lasting four weeks.

### 2.3.2 Trigger for the monitoring of schools

Attack rate reduction is more substantial for larger values of the trigger (Figure S7). However, reduction is negligible if the excess absenteeism threshold is very high, since in that case the threshold in single schools (or classes) is reached only when infection is widespread, regardless of the value of the trigger. This means that for high excess absenteeism thresholds, the outcome of strategies is independent of the number of cases in the population triggering their application.

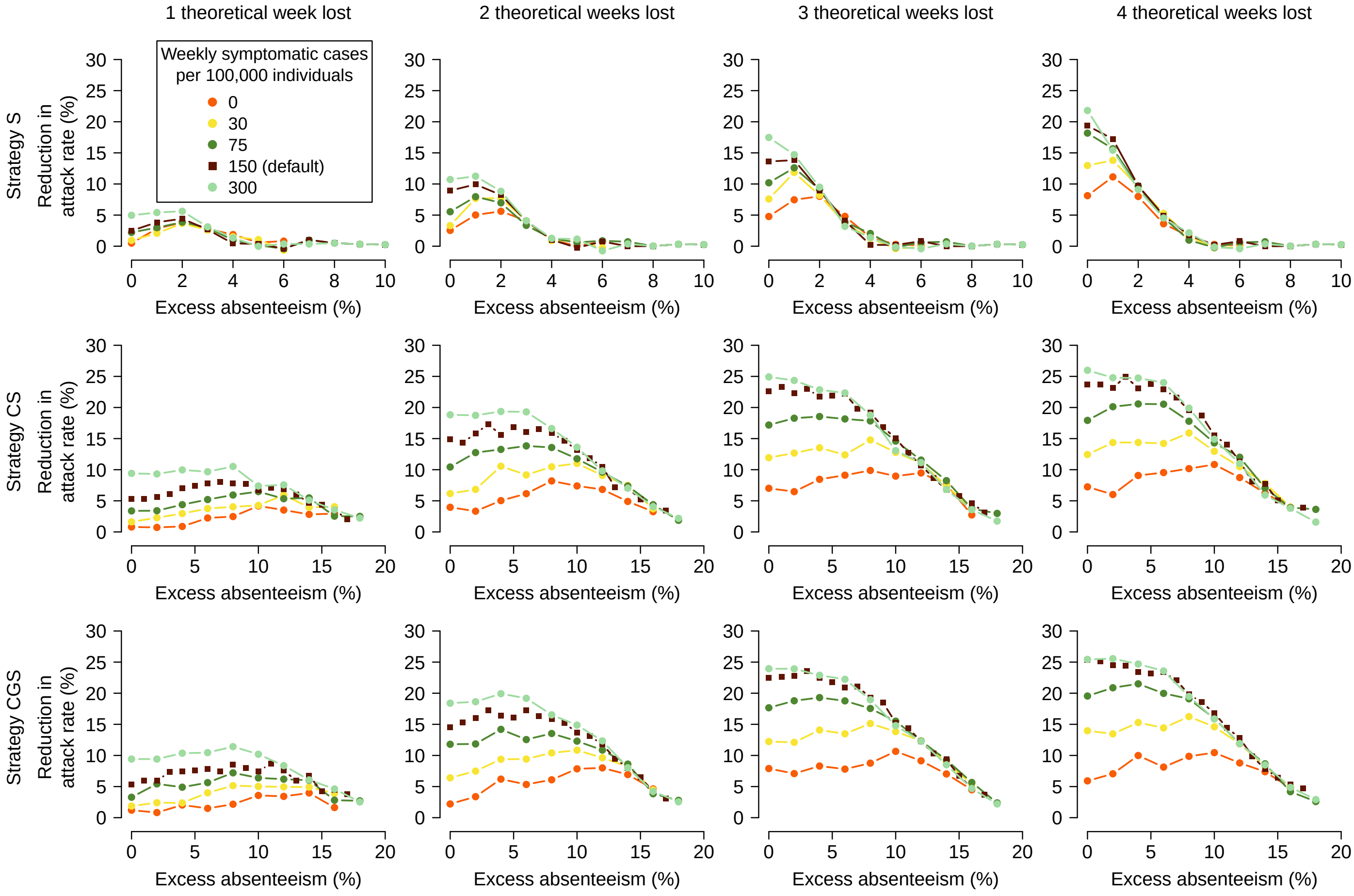


Figure S7: **Attack rate reduction for different triggers of absenteeism monitoring.** The values tested are 0, 30, 75, 150 or 300 new weekly symptomatic cases per 100,000 individuals (see colors and shapes of dots). All remaining parameters take the reference values reported in Tables S1 and S2. Panels refer to the three strategies S, CS and CGS (rows), with closures leading to 1, 2, 3 and 4 theoretical weeks lost (columns).

### 2.3.3 Absenteeism threshold

An alternative way to measure absenteeism is to consider a threshold in terms of absolute instead of excess absenteeism. This has little impact on the outcome (Figure S8): in fact, as the range of attack rate reduction covered by the two options is essentially the same, it is possible to obtain comparable results by choosing thresholds suitably.

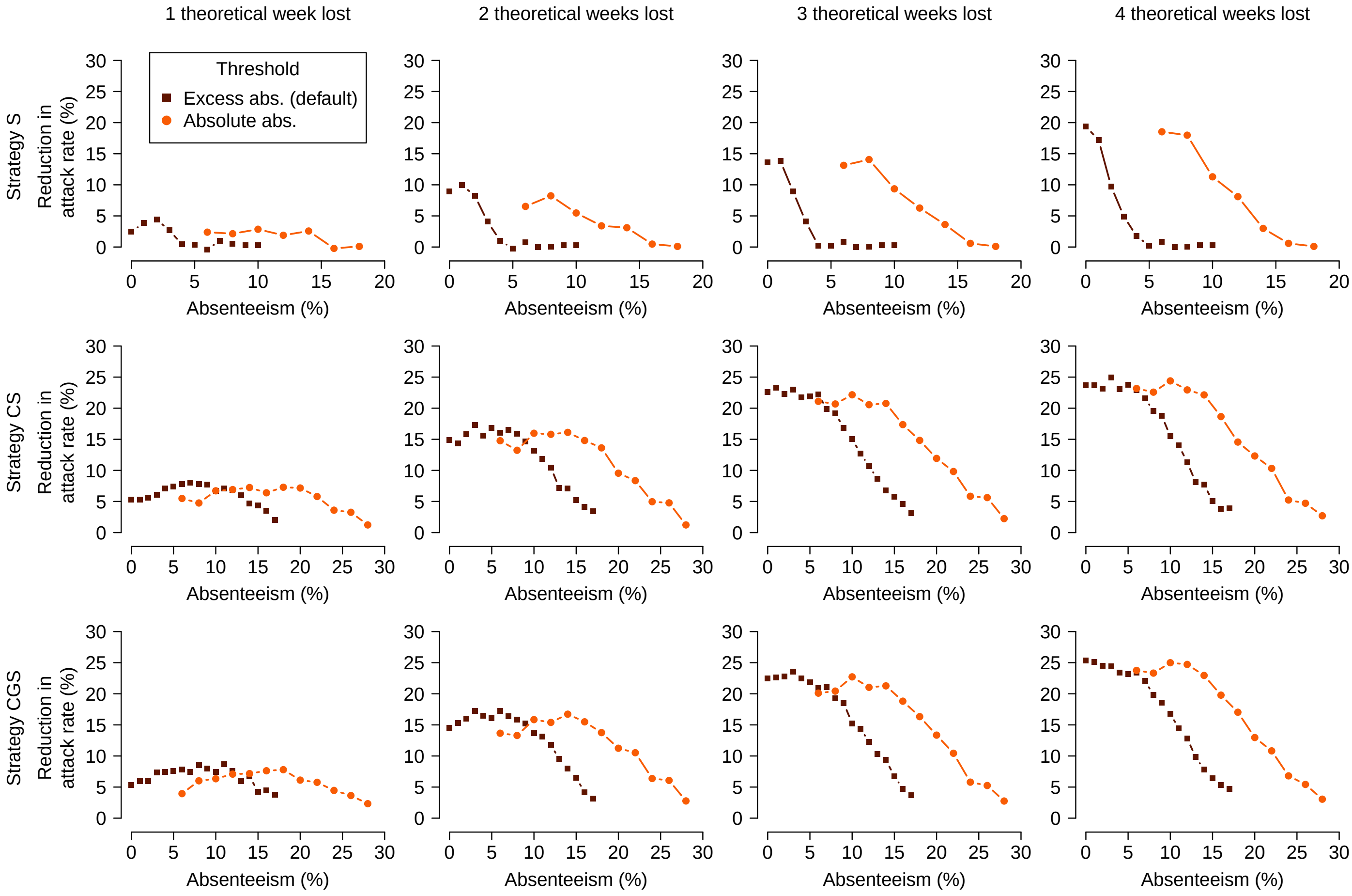


Figure S8: **Attack rate reduction for different measures of absenteeism thresholds.** The absenteeism threshold is computed either as an excess over the physiological rate or as an absolute value. All other parameters take the reference values reported in Tables S1 and S2. Panels refer to the three strategies S, CS and CGS (rows), with closures leading to 1, 2, 3 and 4 theoretical weeks lost (columns).

## 2.4 Sensitivity analysis on epidemiological and behavioral characteristics

The model was calibrated on the epidemiological characteristics of the 2009 influenza pandemic in Italy, and considering the contact patterns that emerged from a study in a French primary school [7]; the corresponding results are presented in the main text. We have also analyzed the effectiveness of school closure strategies under alternative hypotheses for epidemiological and behavioral features. In the following we consider variations in:

1. effective reproduction number;
2. relative susceptibility to infection of adults with respect to underaged individuals;
3. probability of becoming symptomatic upon infection;
4. contact patterns inside schools;
5. relative transmission rate of symptomatic individuals with respect to asymptomatic individuals.

The outcome of school closure strategies in terms of peak week incidence reduction is affected by biological and behavioral characteristics (see Figure S9; effect on attack rate reduction is presented in the main text).

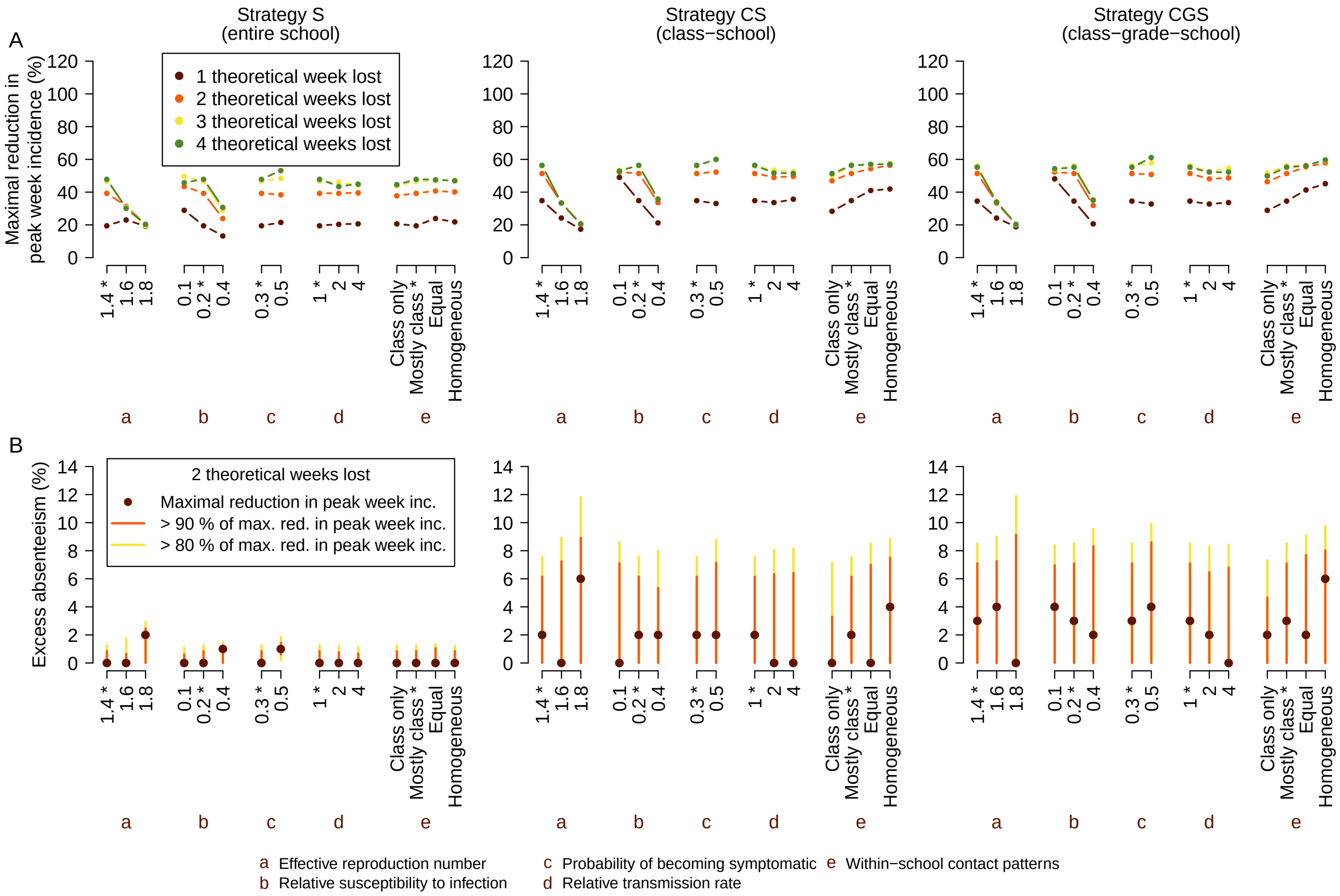


Figure S9: **Sensitivity of peak week incidence to biological and behavioral characteristics.** Unless otherwise stated, parameters take the reference values reported in Table S1. a-e denote the analyzed features. Asterisks indicate the default values. Means are computed over 100 stochastic realizations. **A** Maximal peak week incidence reduction for the strategies with varied biological and behavioral characteristics for 1, 2, 3, and 4 theoretical weeks lost. **B** Ranges of excess absenteeism for which peak week incidence reduction for the strategies with varied biological and behavioral characteristics is at least 90% (red) and 80% (yellow) of the maximal value. Only strategies leading to 2 theoretical weeks lost are considered. Dots indicate where the maximum is attained.

### 2.4.1 Effective reproduction number

We performed model simulations assuming different values for the effective reproduction number (namely 1.6 and 1.8). We observe that, for lower values of the effective reproduction number, strategy S is not able to decrease the attack rate of the epidemic for larger values of the excess absenteeism threshold; on the other hand, when the effective reproduction number is 1.8, the attack rate reduction produced by all strategies is very limited but nearly constant over the excess absenteeism threshold. This behavior can be probably due to the fact that, when the effective reproduction number is low, high values of the excess absenteeism in entire schools cannot be reached, while for large values of the effective reproduction number infection is widepsread in school to such an extent that no absenteeism threshold yields a marked attack rate decrease.

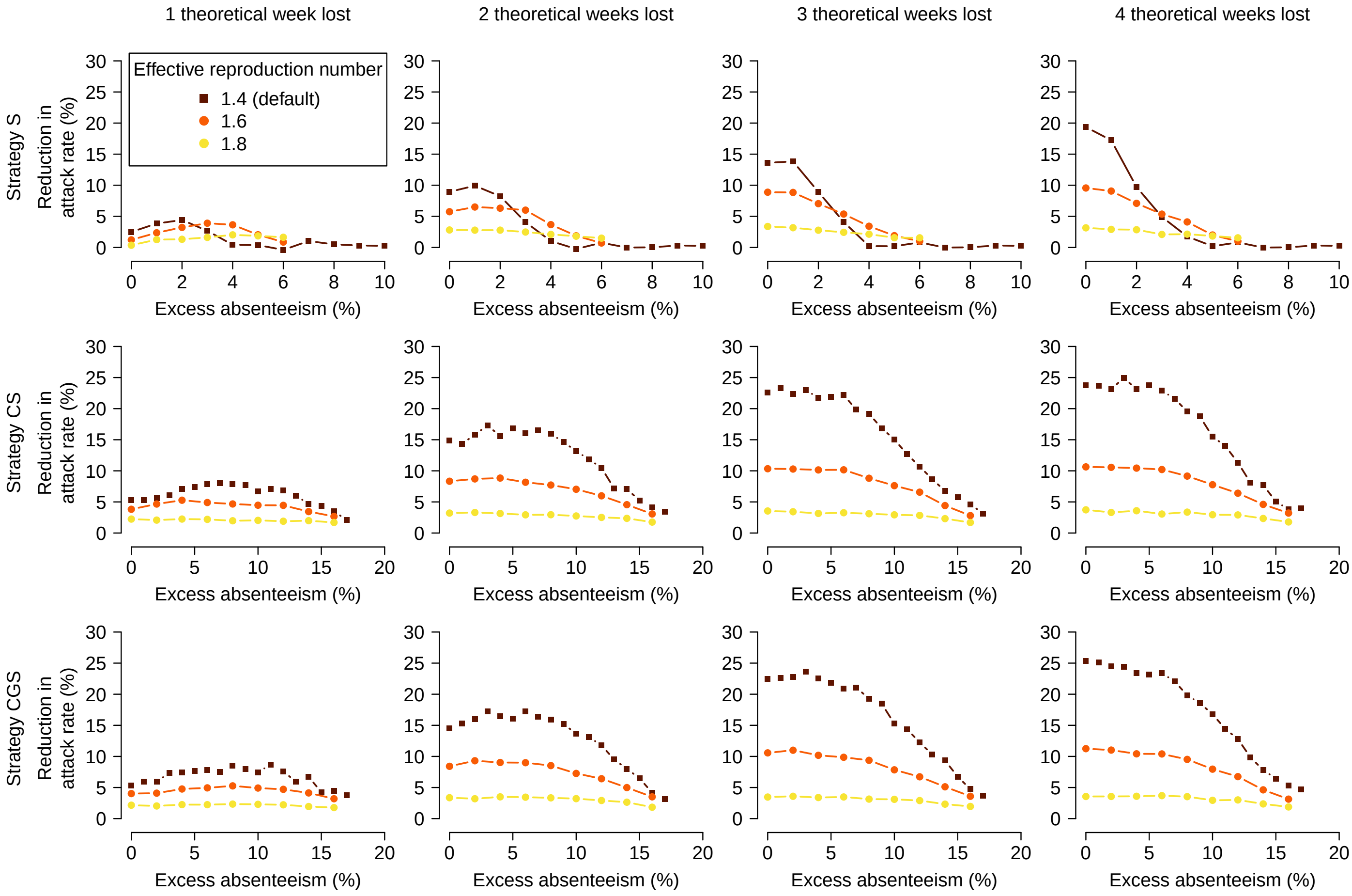


Figure S10: **Attack rate reduction for different values of the effective reproduction number.** Tested values are $\mathcal{R}_e$ = 1.4, 1.6 and 1.8. All remaining parameters take the reference values reported in Tables S1 and S2. Panels refer to the three strategies S, CS and CGS (rows), with closures leading to 1, 2, 3 and 4 theoretical weeks lost (columns).

### 2.4.2 Relative susceptibility to infection

Variations in the relative susceptibility to infection of adults with respect to underaged individuals greatly affect on the efficiency of the strategies (see Figure S11). School closure strategies are more effective if infection affects pupils at a higher rate than adults, since they are aimed at disrupting the network of contacts that pupils have with individuals of similar age. All strategies are most beneficial if the excess absenteeism threshold is low, for all values of the number of theoretical weeks lost.

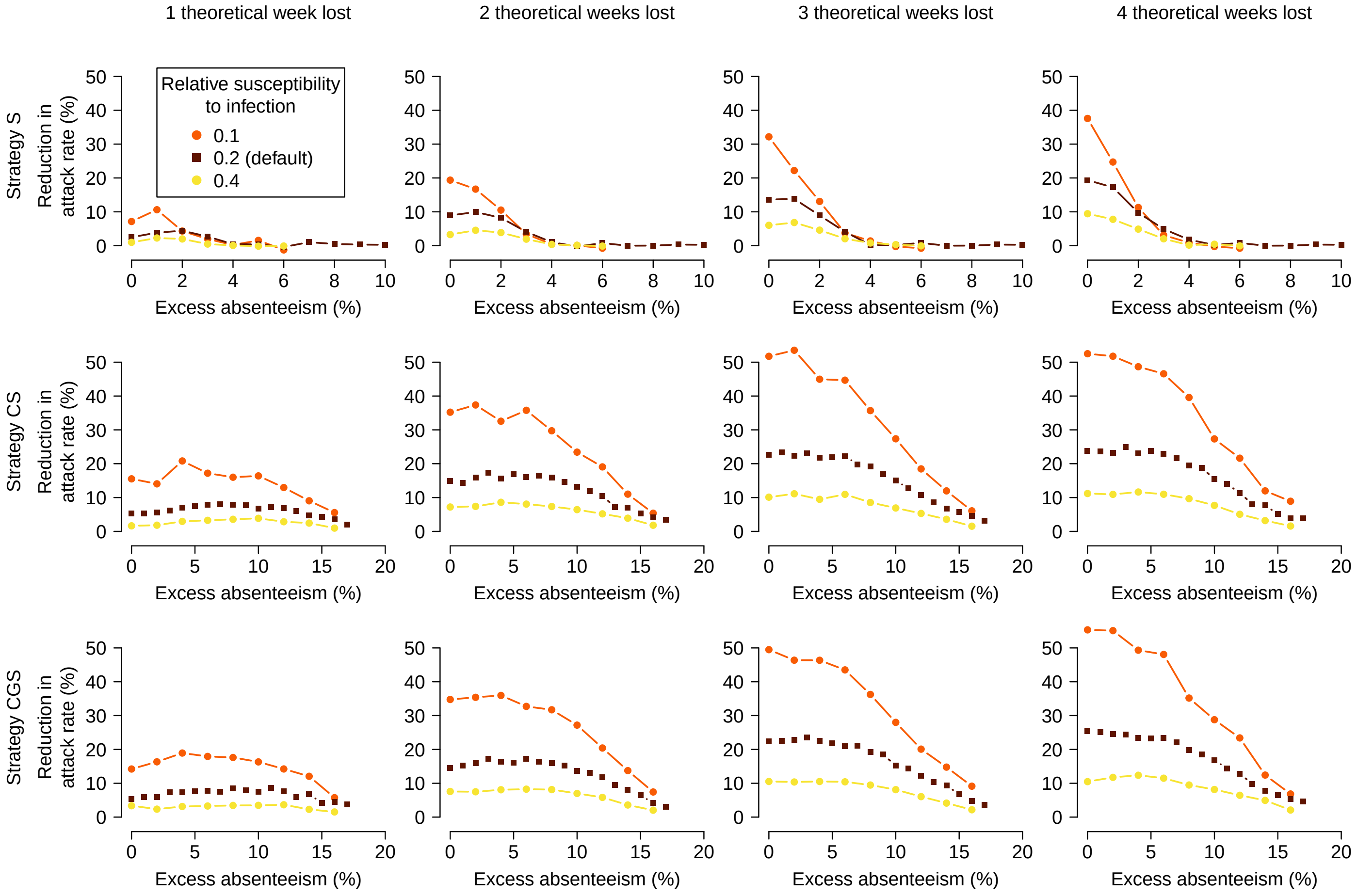


Figure S11: **Attack rate reduction for different values of the relative susceptibility to infection of adults with respect to underaged.** Tested values are 0.1, 0.2 and 0.4. All remaining parameters take the reference values reported in Tables S1 and S2. Panels refer to the three strategies S, CS and CGS (rows), with closures leading to 1, 2, 3 and 4 theoretical weeks lost (columns).

### 2.4.3 Probability of developing symptoms

We investigated values of the probability for an individual infected with influenza to become symptomatic between 30% and 50% [9]. School closure strategies are more efficient if the probability of being symptomatic is higher (see Figures S12): in fact, this implies a larger number of self-isolating individuals, causing an increase of influenza-induced absenteeism that makes unnecessary closures less likely.

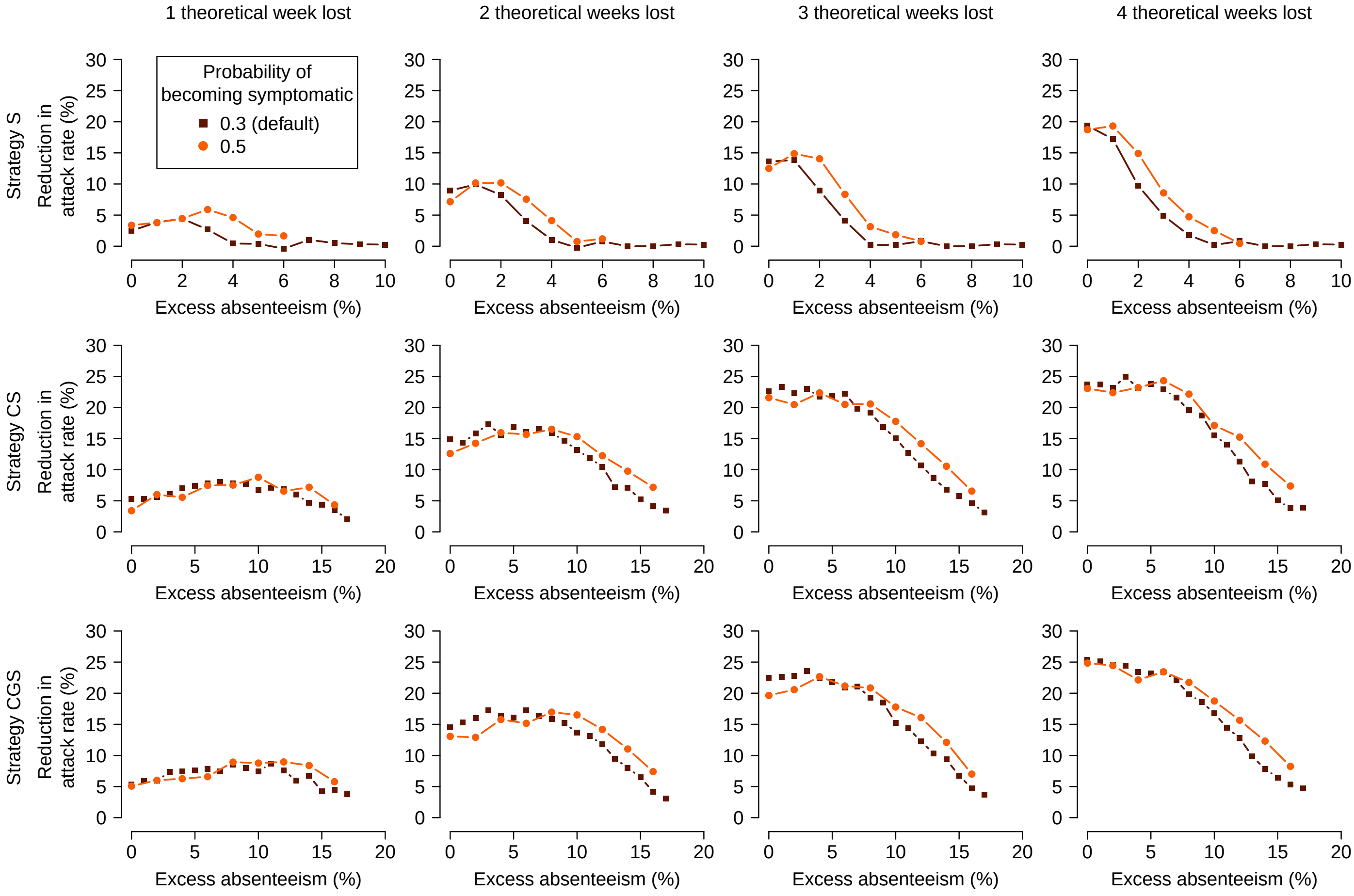


Figure S12: **Attack rate reduction for different values of the probability of developing symptoms.** Tested values are 30% and 50%. All remaining parameters take the reference values reported in Tables S1 and S2. Panels refer to the three strategies S, CS and CGS (rows), with closures leading to 1, 2, 3 and 4 theoretical weeks lost (columns).

### 2.4.4 Relative transmission rate of symptomatic individuals with respect to asymptomatic individuals

The relative transmission rate of symptomatic individuals with respect to asymptomatic individuals has very little influence on school closure strategies, as can be seen in Figure S13.

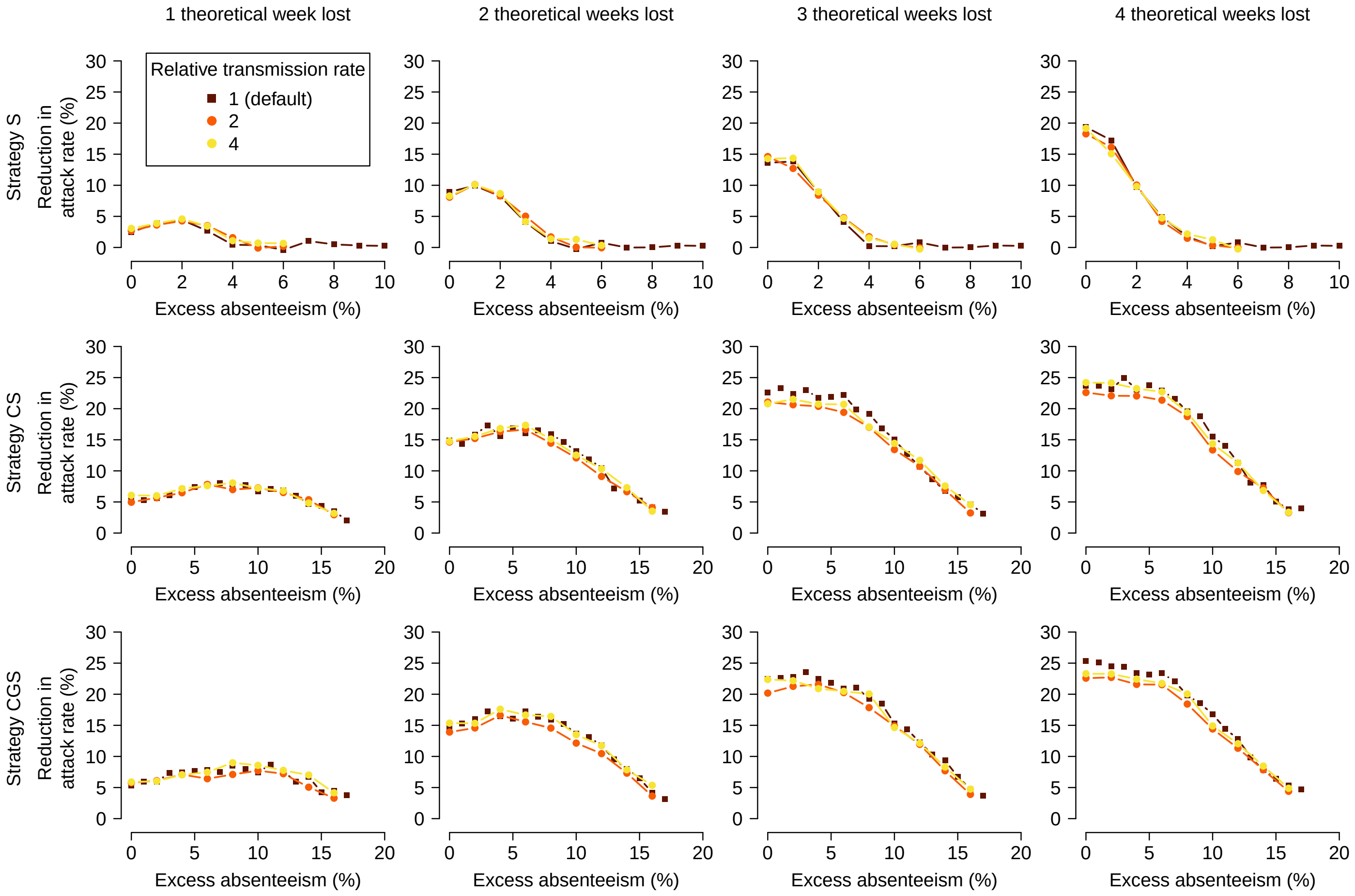


Figure S13: **Attack rate reduction for different values of the relative transmission rate of symptomatic with respect to asymptomatic individuals.** All remaining parameters take the reference values reported in Tables S1 and S2. Panels refer to the three strategies S, CS and CGS (rows), with closures leading to 1, 2, 3 and 4 theoretical weeks lost (columns).

### 2.4.5 Within-school contact patterns

Different hypotheses on within-school contact patterns have an impact on the effectiveness of strategies: if we suppose within-school contacts to be homogeneous, the effectiveness of gradual strategies is greater (see Figure S14): in that case influenza can spread faster, leading to the simultaneous closure of a large number of classes and hence to an early closure of entire schools.

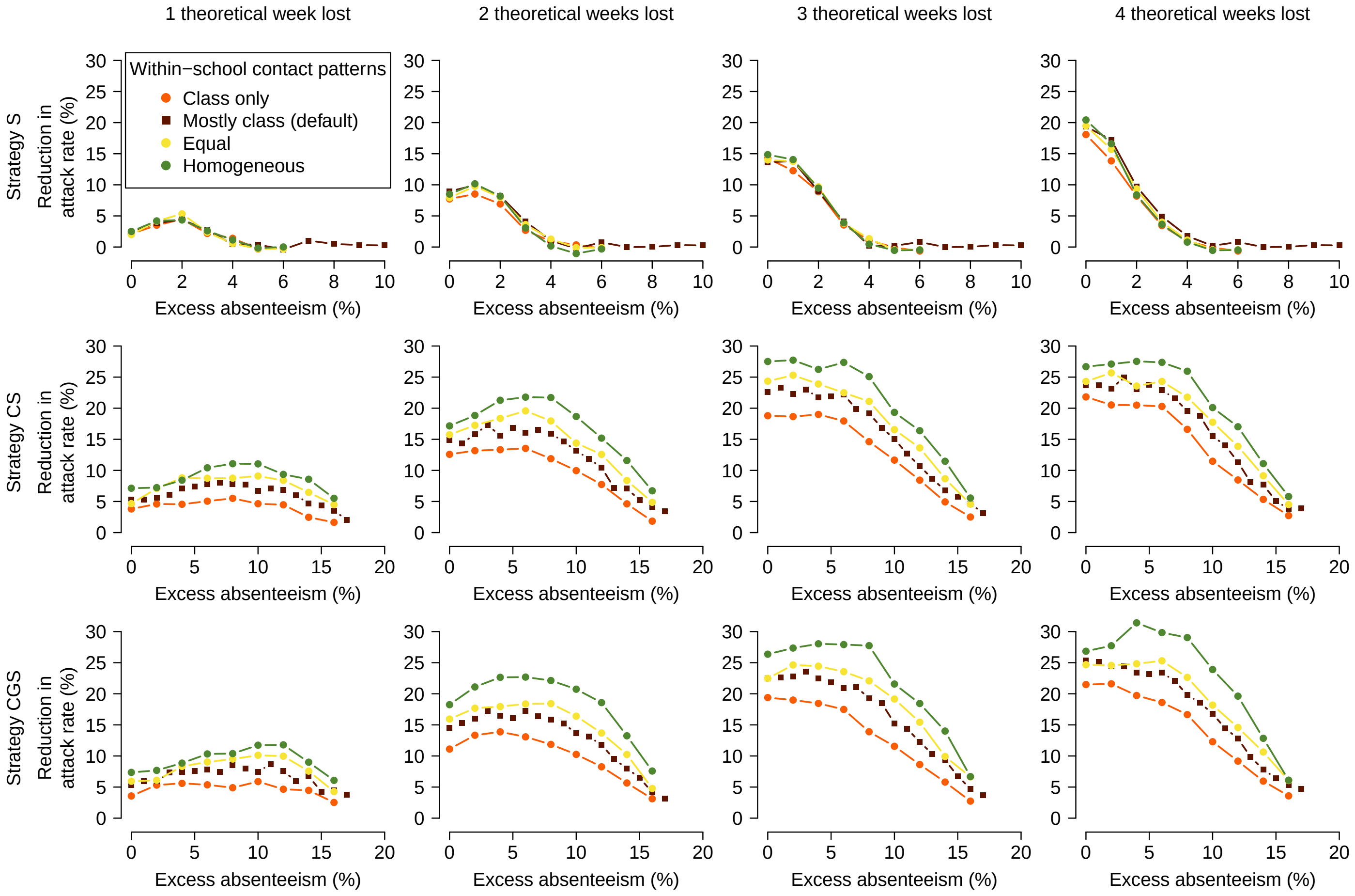


Figure S14: **Attack rate reduction for different distributions of contacts inside schools**. We tested the options 'mostly class' (class 72.6%, remaining grade 9.9%, remaining school 17.5%), 'class only' (100%, 0%, 0%), 'equal' (33%, 33%, 33%), 'homogeneous' (over the entire school). All remaining parameters take the reference values reported in Tables S1 and S2. Panels refer to the three strategies S, CS and CGS (rows), with closures leading to 1, 2, 3 and 4 theoretical weeks lost (columns).

# References


[1] Ajelli M, Poletti P, Melegaro A, Merler S (2014) The role of different social contexts in shaping influenza transmission during the 2009 pandemic. Sci Rep 4: 7218.

[2] Organisation for Economic Co-operation and Development (OECD) (2014). Education at a Glance 2014: OECD Indicators. URL http://www.oecd.org/edu/Education-at-a-Glance-2014.pdf.

[3] Organisation for Economic Co-operation and Development (OECD) (2014). TALIS 2013 Results. An International Perspective on Teaching and Learning. URL http://dx.doi.org/10.1787/9789264196261-table110-en.

[4] Eurydice-EUROSTAT (2005). Key Data on Education in Europe 2005. URL http://eacea.ec.europa.eu/education/eurydice.

[5] Eurydice-EUROSTAT (2012). Key Data on Education in Europe 2012. URL http://eacea.ec.europa.eu/education/eurydice.

[6] Italian National Institute of Statistics (ISTAT) (2010). Uso del Tempo. URL http://www.istat.it/it/archivio/6619. (In Italian).

[7] Gemmetto V, Barrat A, Cattuto C (2014) Mitigation of infectious disease at school: targeted class closure vs school closure. BMC Infect Dis 14: 695.

[8] Lessler J, Reich NG, Brookmeyer R, Perl TM, Nelson KE, et al. (2009) Incubation periods of acute respiratory viral infections: a systematic review. Lancet Infect Dis 9: 291–300.

[9] Ferguson NM, Cummings DA, Cauchemez S, Fraser C, Riley S, et al. (2005) Strategies for containing an emerging influenza pandemic in Southeast Asia. Nature 437: 209–214.

[10] Cowling BJ, Fang VJ, Riley S, Peiris JM, Leung GM (2009) Estimation of the serial interval of influenza. Epidemiology 20: 344-347.

[11] Merler S, Ajelli M, Pugliese A, Ferguson NM (2011) Determinants of the Spatiotemporal Dynamics of the 2009 H1N1 Pandemic in Europe: Implications for Real-Time Modelling. PLoS Comput Biol 7: e1002205.

[12] Cauchemez S, Bhattarai A, Marchbanks TL, Fagan RP, Ostroff S, et al. (2011) Role of social networks in shaping disease transmission during a community outbreak of 2009 H1N1 pandemic influenza. Proc Natl Acad Sci USA 108: 2825–2830.

[13] CDC Centers for Disease Control and Prevention (2009). CDC Recommendations for the Amount of Time Persons with Influenza-Like Illness Should be Away from Others. URL http://www.cdc.gov/h1n1flu/guidance/exclusion.htm.

[14] Wallinga J, Lipsitch M (2007) How generation intervals shape the relationship between growth rates and reproductive numbers. P Roy Soc Lond B Bio 274: 599–604.

[15] Merler S, Ajelli M, Camilloni B, Puzelli S, Bella A, et al. (2013) Pandemic influenza A/H1N1pdm in Italy: age, risk and population susceptibility. PloS One 8: e74785.

[16] Hayward AC, Fragaszy EB, Bermingham A, Wang L, Copas A, et al. (2014) Comparative community burden and severity of seasonal and pandemic influenza: results of the Flu Watch cohort study. Lancet Resp Med 2: 445–454.
